# Acute Myeloid Leukemia Cells Educate Mesenchymal Stromal Cells toward an Adipogenic Differentiation Propensity with Leukemia Promotion Capabilities

**DOI:** 10.1002/advs.202105811

**Published:** 2022-03-20

**Authors:** Luwen Zhang, Qiong Zhao, Hui Cang, Ziqiang Wang, Xiaojia Hu, Ruolang Pan, Yang Yang, Ye Chen

**Affiliations:** ^1^ Department of Genetics, and Department of Genetic and Metabolic Disease The Children's Hospital, Zhejiang University School of Medicine, National Clinical Research Center for Child Health Hangzhou Zhejiang 310052 China; ^2^ Zhejiang Provincial Key Laboratory of Genetic and Developmental Disorders Institute of Genetics, Zhejiang University Hangzhou Zhejiang 310058 China; ^3^ Zhejiang Provincial Key Laboratory of Cell‐Based Drug and Applied Technology Development Institute for Cell‐Based Drug Development of Zhejiang Province S‐Evans Biosciences Hangzhou Zhejiang 310023 China; ^4^ Bone Marrow Transplantation Center, Institute of Hematology, The First Affiliated Hospital Zhejiang University School of Medicine Hangzhou Zhejiang 310004 China

**Keywords:** acute myeloid leukemia (AML), cell‐derived exosomes, mesenchymal stromal cells (MSCs)

## Abstract

Mesenchymal stromal cells (MSCs) are essential elements of the bone marrow (BM) microenvironment, which have been widely implicated in pathways that contribute to leukemia growth and resistance. Recent reports showed genotypic and phenotypic alterations in leukemia patient‐derived MSCs, indicating that MSCs might be educated/reprogrammed. However, the results have been inconclusive, possibly due to the heterogeneity of leukemia. Here, the authors report that acute myeloid leukemia (AML) induces MSCs towards an adipogenic differentiation propensity. RNAseq analysis reveal significant upregulation of gene expression enriched in the adipocyte differentiation process and reduction in osteoblast differentiation. The alteration is accompanied by a metabolic switch from glycolysis to a more oxidative phosphorylation‐dependent manner. Mechanistic studies identify that AML cell‐derived exosomes play a vital role during the AML cell‐mediated MSCs education/reprogramming process. Pre‐administration of mice BM microenvironment with AML‐derived exosomes greatly enhance leukemia engraftment in vivo. The quantitative proteomic analysis identified a list of exosomal protein components that are differently expressed in AML‐derived exosomes, which represent an opportunity for novel therapeutic strategies based on the targeting of exosome‐based AML cells‐MSCs communication. Collectively, the data show that AML‐educated MSCs tend to differentiate into adipocytes contributing to disease progression, which suggests complex interactions of leukemia with microenvironment components.

## Graphical abstract

Acute myeloid leukemia (AML) cells induce alteration of regular mesenchymal stromal cells (MSCs) toward an adipogenic differentiation propensity accompanied by a metabolic switch from glycolysis to more oxidative phosphorylation‐dependent manner, which in turn contributes to leukemia progression. Exosomes can be critical mediators of the AML cells‐mediated MSCs education. These findings suggest complex interactions of leukemia with microenvironment components.

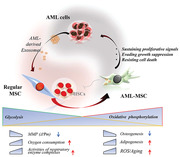

## 1 Introduction

Multipotent mesenchymal stem/stromal cells (MSCs) are self‐renewing and multipotent progenitors that can differentiate into various cell types, such as osteoblasts, adipocytes, and endothelial cells, which constitute the main cellular compartment of bone marrow (BM) niche.^[^ ^1^ ^]^ MSCs in the BM niche produce various cytokines/chemokines to regulate signaling that support self‐renewal and differentiation of hematopoietic stem cells (HSCs).^[^ ^2^ ^]^ Recent studies suggested that leukemia cells infiltrate these BM niches and may hijack the homeostatic mechanisms of HSCs.^[^ ^3^ ^]^ There is growing evidence supporting the theory that MSCs play a pivotal role in leukemia progression and development of chemoresistance.^[^ ^3^, ^4^ ^]^ In addition, MSCs from leukemia patients frequently carry genomic alterations that may contribute to the pathogenesis of disease.^[^ ^5^ ^]^ Mouse studies also proved that specific modification in MSCs alone can initiate leukemia.^[^ ^6^ ^]^ Currently, there is increasing interest in targeting MSCs for therapeutic applications in leukemia patients.

Growing in‐depth studies in the last decade have shown dynamic and sophisticated cooperation between MSCs and leukemic cells.^[^ ^7^ ^]^ Other groups and we have reported that leukemia patient‐derived MSCs were genetically and functionally different from regular healthy donor‐derived MSCs.^[^ ^8^ ^]^ Multiple results also suggested the participation of MSC reprogramming in the pathogenesis or progression of hematological malignancies. For example, Ghosh et al. showed that B‐cell chronic lymphocytic leukemia (CLL) cells directly delivered phospho‐receptor tyrosine kinase Axl to MSCs in association with AKT activation;^[^ ^9^ ^]^ Corrado et al. showed that chronic myeloid leukemia (CML) cells stimulate MSCs to produce IL 8 that, in turn, to affect leukemia cell progression both in vitro and in vivo;^[^ ^10^ ^]^ Horiguchi et al. found that extracellular vesicle miR‐7977 derived from acute myeloid leukemia/myelodysplastic syndromes (AML/MDS) cells were transferred into MSCs and thus impair the stem cell‐supporting capacity.^[^ ^11^ ^]^ Despite clear evidence for the existence of these interactions, the precise repercussions on the reprogramming of MSCs are still poorly understood. Further, there were inconsistent findings of the leukemic cell‐mediated functional alterations in MSCs. Liu et al. reported that multiple myeloma cells inhibited the osteoblastic differentiation of MSCs;^[^ ^12^ ^]^ studies reported by Le et al.^[^ ^13^ ^]^ and Azadniv et al.^[^ ^14^ ^]^ suggested increased propensity toward adipogenesis of BM microenvironment in AML, while Battula et al. showed AML cells induced MSCs to undergo osteogenic differentiation.^[^ ^15^ ^]^ Nevertheless, researchers now reach a consensus that leukemic cells educate MSCs to further promote leukemia progression and survival.

In this work, we used both in vitro and in vivo models to identify changes in AML related MSCs and discovered that AML cells educate MSCs toward an adipogenic differentiation propensity with a metabolic switch from glycolysis to more oxidative phosphorylation (OXPHOS)‐dependent manner. Exosomes produced by AML cells were at least partially responsible for the AML cell‐mediated MSCs reprogramming process. Syngeneic mouse AML model also confirmed that AML cell‐derived exosomes alter the bone marrow MSCs to facilitate AML engraftment and progression.

## 2 Results

### 2.1 AML Patient‐Derived MSCs Exhibited Increased Adipogenic and Delayed Osteogenic Differentiation Capacities

**Figure 1:**
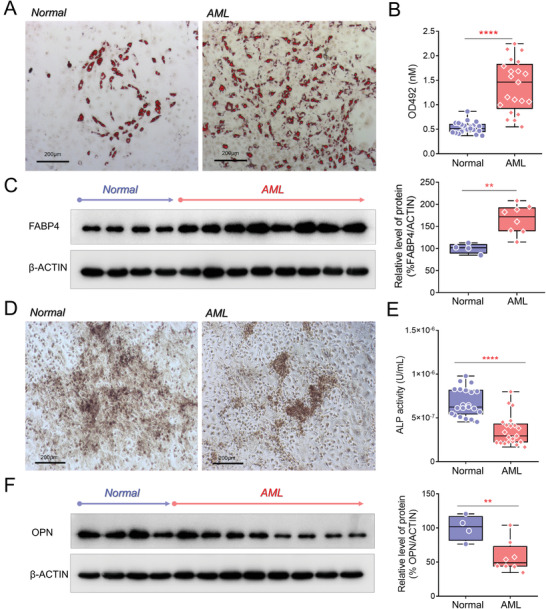
AML patient‐derived MSCs exhibited increased adipogenic and delayed osteogenic differentiation capacities. A) Normal and AML‐derived hMSCs were induced for adipocytic lineage differentiation in a specific medium. Oil Red O cytochemical lipid staining (red) was performed on day 14, revealing a significantly increased number of differentiated adipocytes from AML‐hMSCs. B) The Oil Red O staining was further extracted and determined in a microplate reader at 492 nm. C) Western blot analysis confirmed the increased adipogenic potential of AML‐hMSCs with an enhanced level of FABP4 detected in differentiated adipocytes. D) Normal and AML‐derived hMSCs were differentiated in osteogenesis medium for 21 d and were positive for ALP staining. The decreased osteogenetic potential of AML‐hMSCs was confirmed by both E) ALP activity analysis and F) semiquantitative protein assay for OPN. *β*‐ACTIN was used as reference housekeeping control. (Unpaired, two‐tailed Student's, *n* ≥ 3, mean with SD, *p < 0.05, **p < 0.01, ***p < 0.001, ****p < 0.0001).

Human MSCs were isolated from the BM of eight AML‐M4 patients (AML‐hMSCs) and four age‐matched regular healthy donors (N‐hMSCs) (Table S1, Supporting Information). Both types of cells possessed normal viability, with the fraction of dead/dying cells being less than 5%, and conformed to the minimal criteria of MSC defined by the International Society for Cellular Therapy. Briefly, >99% of the cells were positive for anti‐CD73, anti‐CD90, and anti‐CD105 staining but absent for CD14, CD19, CD34, CD45, and HLA‐DR. In addition, the cells can successfully differentiate into classical mesenchymal derivatives, including osteoblasts, adipocytes, and chondrocytes. Consistent with previous studies,^[^ ^8^, ^13^ ^]^ we found that the two types of MSCs had significant differences in adipogenic and osteogenic differentiation ability. We cultured AML‐hMSCs and N‐hMSCs in 24‐well plates in adipogenic or osteogenic differentiation medium to extend these observations. As shown in **Figure** **1**A, representative images revealed a significantly increased number of differentiated adipocytes from AML‐hMSCs on day 14 of differentiation; moreover, the Oil Red O staining was extracted in isopropanol and determined in a microplate reader at 492 nm, which was ≈2.6‐fold higher in AML‐hMSCs group (Figure 1B). These findings were further confirmed using western blot analysis of FABP4 protein levels in differentiated cells (Figure 1C). There was an ≈1.7‐fold increase in the expression of FABP4 in differentiated AML‐MSCs compared to normal controls (*P <* 0.005). Similarly, as visualized by alkaline phosphatase (ALP) staining (Figure 1D), AML‐hMSCs exhibited delayed differentiation on day 21 compared to the N‐hMSCs. Quantitative analysis showed a ∼0.48‐fold decrease of ALP activities in differentiated AML‐hMSCs (*P* < 0.001). Western blot assays revealed an ≈0.42‐fold reduction of OPN expression levels in differentiated AML‐MSCs (P<0.005) to prove the finding further. Thus, we confirmed differences between AML‐hMSCs and N‐hMSCs' differentiation potential and proposed that AML cells might be responsible for this alteration in MSCs.

### 2.2 In Vitro AML Cell Incubation Facilitated Normal hMSCs to Differentiate into the Adipogenic Lineage and Retarded the Osteogenic Differentiation

To prove our hypothesis, we adopted an in vitro transwell coculture system to see whether the alteration in MSCs could be reproduced. First, N‐hMSCs at passage 3 were cocultured with fresh AML blasts for nine days using a Transwell system and then induced for adipogenic or osteogenic differentiation. As shown in Figure S1A,B (Supporting Information), coculture with AML blasts did affect the differentiation capabilities of N‐hMSCs. The differences of Oil Red O staining (≈1.6‐fold increase, *P* < 0.05) and ALP activities (≈0.24‐fold decrease, *P* < 0.05) were significant; however, there were considerable variations of the effects among different AML samples.

**Figure 2:**
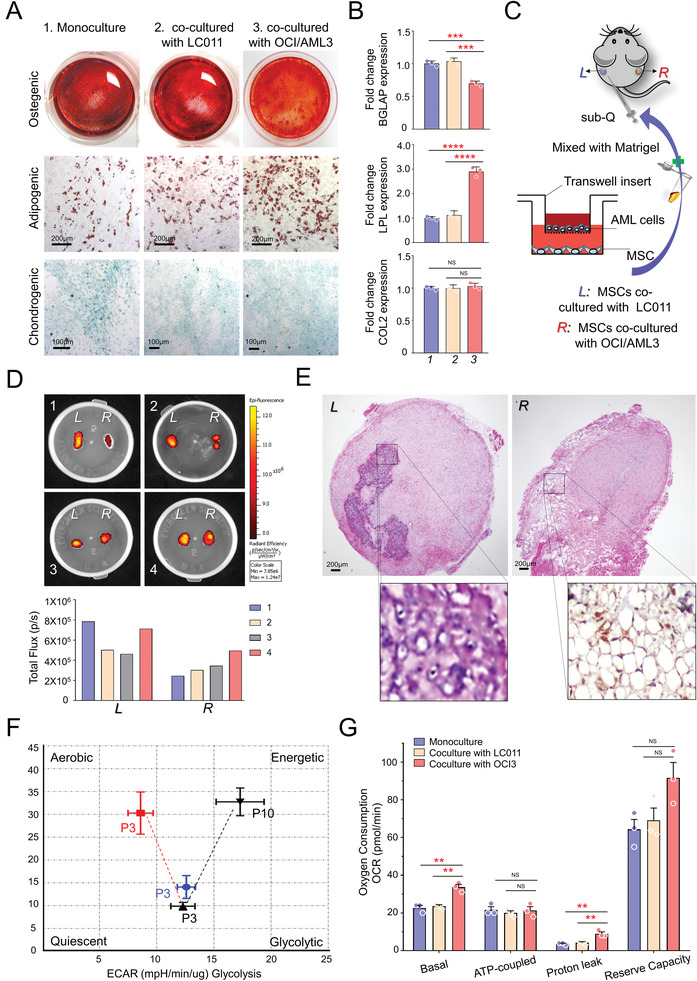
AML cell coculture facilitated normal hMSCs to differentiate into the adipogenic lineage and retarded the osteogenic differentiation. A) N‐hMSCs with/without coculture were induced to differentiate along adipogenic, osteogenic, and chondrogenic lineages. B) The differentiation degrees were evaluated by qRT‐PCR analysis of lineage marker gene expression. *GAPDH* was used as a housekeeping gene. All measures were performed per triplicate. C) Schema of in vivo MSC differentiation study. N‐hMSCs pre‐cocultured with either OCI/AML3 or LC011 cells were injected subcutaneously into the opposite flanks of NOD/SCID mice with Matrigel (*n* = 4). These cells developed into bone‐like cells tissues with osteoblastic activity in six weeks. D) Mice were intravenously injected with OsteoSense750 and sacrificed in 24 h. These differentiated implants were imaged using the IVIS system, and the fluorescent signals were calculated. E) Representative H&E staining images showed more osteoblastic differentiation in the LC011‐cocultured group (L, the insert exhibits typical bone structure and osteoblasts); on the other hand, OCI/AML3‐cocultured hMSCs develop into a higher ratio of adipocytes (R, the insert exhibits immunohistochemical staining using PPARg antibody). F) Seahorse XF cell energy phenotype tests were performed (*n* = 5 for each cell type): N‐hMSCs at early passage (black dot, P3), N‐hMSCs at late passage (black dot, P10), N‐hMSCs cocultured with OCI/AML3 (red dot, P3), N‐hMSCs cocultured with LC011 (blue dot, P3). G) Mitochondrial respiration parameters were calculated after the Seahorse XF Cell Mito Stress test. Bar: 200 µm (one‐way ANOVA, mean with SD, *p < 0.05, **p < 0.01, ***p < 0.001, ****p < 0.0001).

To provide further insight regarding the alteration of MSCs during AML cell incubation, we cocultured N‐hMSCs with a standard AML cell line OCI/AML3, which was established from a 57‐year‐old man with acute myeloid leukemia (AML FAB M4). LC011, an age‐matched male healthy volunteer‐derived immortalized lymphocyte cell line, was used as a standard control for coculture experiments. N‐hMSCs (P3) were cocultured with OCI/AML3 or LC011 cells for nine days using a Transwell system, then in vitro tri‐lineage differentiation was induced (**Figure** **2**A). As expected, OCI/AML3‐cocultured N‐hMSCs exhibited lower osteoblastic differentiation but higher adipogenic differentiation potential than hMSCs in control groups. The differentiation degrees were confirmed by qRT‐PCR analysis of lineage marker gene expression (Figure 2B). To further determine the in vivo differentiation capacities, hMSCs cocultured with either OCI/AML3 or LC011 cells were mixed with Matrigel and injected subcutaneously into the opposite flanks of NOD/SCID mice (Figure 2C). The mice were intravenously injected with a bisphosphonate‐derivatized NIRF imaging agent (OsteoSense, excitation 750) six weeks later and sacrificed in twenty‐four hours. The differentiated implants were harvested for fluorescent imaging using the IVIS system. Consistent with our previous findings in vitro, reduced fluorescent signals, representing osteoblast activity, were found in OCI/AML3‐cocultured implants (Figure 2D), suggesting that these MSCs differentiate poorly into the osteoblast lineage in vivo compared to the LC011 cells‐cocultured N‐hMSCs. The histologic analysis further revealed more bone structure in the LC011‐cocultured group, while the OCI/AML3‐cocultured group developed a higher percentage of adipocytes (Figure 2E).

Accumulating evidence has suggested that the regulation of metabolism is essential for MSC differentiation and lineage determination.^[^ ^16^ ^]^ Here we investigated the metabolic changes in N‐hMSCs before and after cocultured with OCI/AML3 or LC011 cells. Cell energy phenotype tests (Figure 2F) revealed that low passage (P3) N‐hMSCs exhibit low levels of mitochondrial and glycolytic activities. Notably, OCR/ECAR ratio at the basal conditions was significantly high in OCI/AML3‐cocultured N‐hMSCs, indicating these N‐hMSCs started using OXPHOS more than glycolysis during energy production. No significant changes were found in LC011‐cocultured N‐hMSCs. Long‐term cultured N‐hMSCs (P10) were used as an aging control, which exhibited a more energetic cell metabolic phenotype. Sequential administration of ETC complex inhibitors during the Seahorse experiments allows for real‐time assessment of different parameters associated with mitochondrial respiration. For example, oligomycin determines the amount of ATP production is linked to respiration; FCCP enables measurement of maximal respiratory capacity, while a combination of rotenone and antimycin A determines the reserved respiratory capacity. The tests showed comparable mitochondrial respiration parameters in the LC011‐cocultured group and monoculture control; on the other hand, OCI/AML3‐cocultured N‐hMSCs exhibited significant increases in basal respiration, proton leak, as well as reserve capacity (Figure 2G).

### 2.3 In Vitro AML Cell Incubation Induced Significant Changes in Gene Profiling in N‐hMSCs

**Figure 3:**
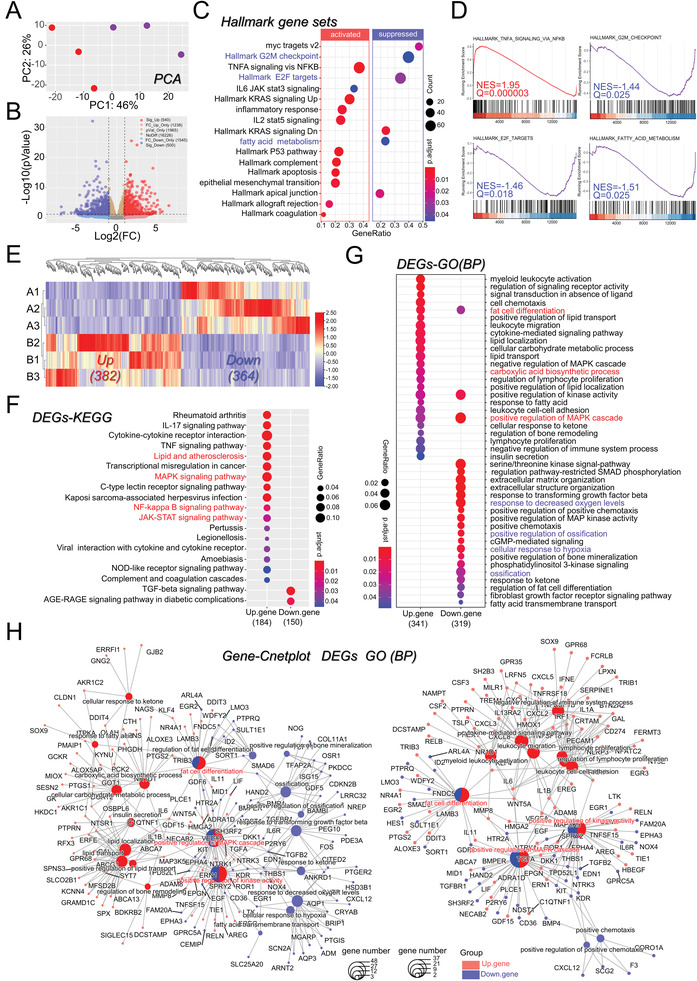
RNA‐seq analysis revealed significant changes in gene expressions in AML‐cocultured MSCs. A) The principal component (PC) of transcription differences shows significant differences between two groups of hMSCs cocultured with AML‐cocultured or LC011. B) Volcano plot illustration of differentially expressed genes (DEGs). Red and blue plots indicate upregulated and downregulated DEGs, respectively. C,D) 15688 protein‐encoding genes were obtained from the corrected FC for enrichment analysis of classic pathways under GSEA. Results showed significantly enriched gene sets in myc targets, G2M checkpoint, TNFA signaling via NFKB and fatty acid metabolism. E) Circular heatmap of 746 differential expressed protein‐coding genes between two groups of MSCs. F) The significant KEGG pathways in the enrichment analysis of DEGs. G) The most significantly regulated GO annotations of BP in the enrichment analysis of the DEGs. H) Network plot of enriched terms.

Having clarified the alternated cellular properties in OCI/AML3‐cocultured N‐hMSCs, we were curious about the differences at the molecular level. RNA‐seq analysis was performed. As shown in **Figure** **3**A, principal component analysis (PCA) of mRNA expression data shows the significant differences between two groups of hMSCs (*n* = 3 in each group). A total of 22 014 genes were detected, including 540 and 500 genes that were significantly upregulated and downregulated (*p* < 0.05, |log_2_FC| > 1) in AML‐cocultured hMSCs compared with LC011‐cocultured hMSCs (Figure 3B). The original FC data of 15 688 protein‐encoding genes were corrected by lfcshrink (Figure S2A, Supporting Information), and Gene Set Enrichment Analysis (GSEA) was conducted. GSEA figured out specific terms activated or suppressed in AML‐cocultured hMSCs (Figure 3C and Figure S2B, Supporting Information), including G2M checkpoint, TNFA signaling via NFKB, E2F targets, fatty acid metabolism, etc. (Figure 3D). Network plots of the screened hallmark terms were illustrated (Figure S2C, Supporting Information). Simultaneously, 746 differentially expressed protein‐coding genes were further screened (Figure 3E) for downstream analyses, including 382 upregulated and 364 downregulated in AML‐cocultured MSCs. The KEGG analysis of these 746 DEGs (Figure 3F and Figure S3, Supporting Information) was consistent with our findings by GSEA.

Furthermore, gene ontology (GO) analysis (biological process, BP) of the 746 DEGs indicated those upregulated ones in AML‐cocultured hMSCs were involved with myeloid leukocyte activation, fat cell differentiation, leukocyte migration, cell carbohydrate metabolic process, and regulation of bone remodeling (Figure S4, Supporting Information). Instead, the significantly downregulated DEGs were involved with extracellular matrix organization, extracellular structure organization, response to decreased oxygen levels, etc. (Figure 3G). Network plots of DEGs with enriched terms were illustrated using R studio (Figure 3H). Next, the protein–protein interaction (PPI) networks of DEGs were drawn using the STRING online platform; the expression and network of top 20 upregulated and downregulated hub genes were visualized (Figure S5, Supporting Information).

**Figure 4:**
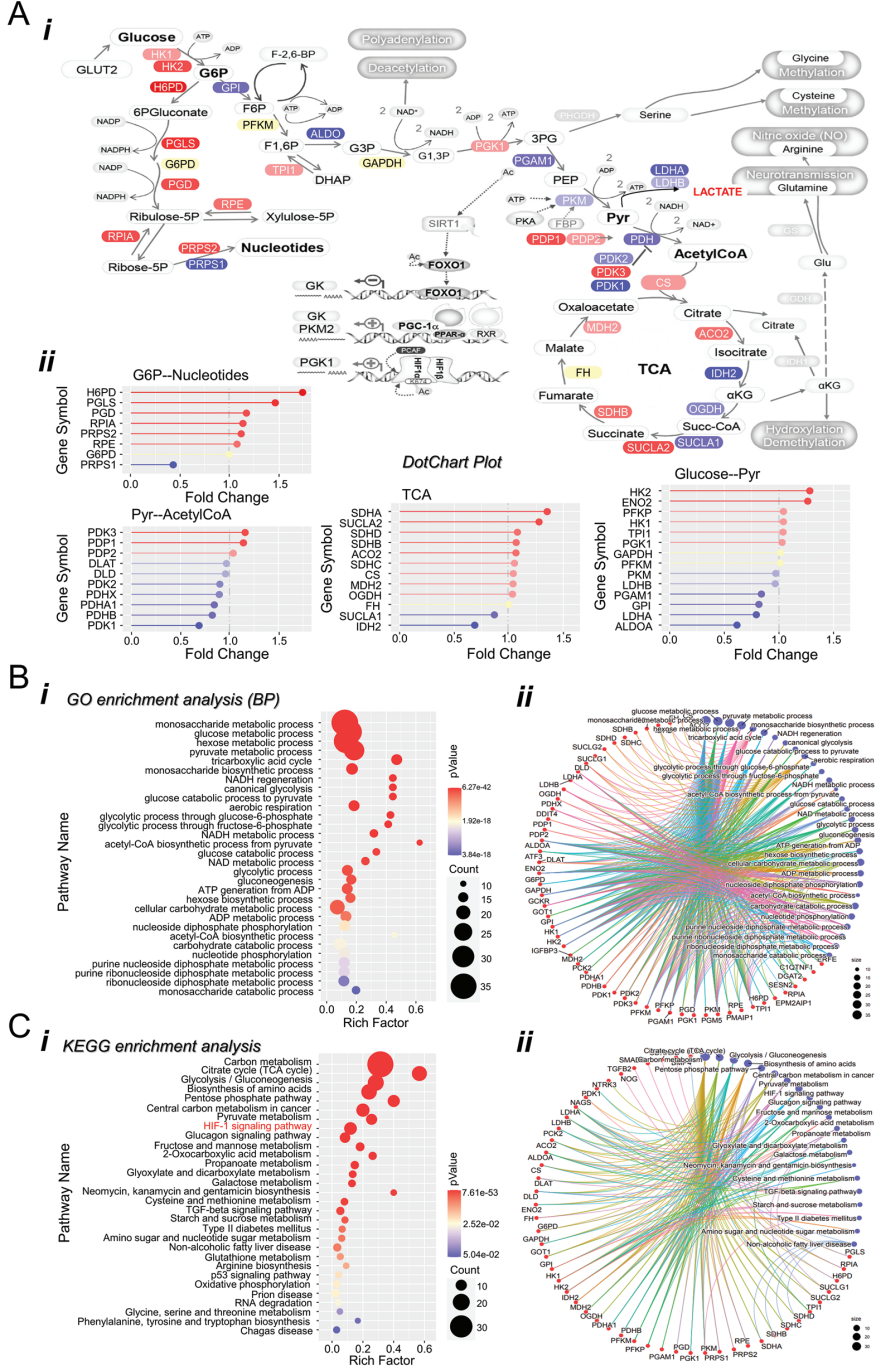
AML‐cocultured N‐hMSCs exhibit alternated gene expression terms related to the cellular carbohydrate metabolic process. A) RNA‐seq data revealed transcriptional regulation of metabolic genes: (i) representative genes were indicated in the metabolic diagram; (ii) the FPKM values of metabolic genes are shown as fold change (AML‐cocultured N‐hMSCs/control N‐hMSCs). B,C) GO (BP) and KEGG enrichment analysis results. Bi,Ci) Bubble charts show the enrichment analysis results of biological processes and KEGG pathways Bii,Cii) Selected pathways (*P* < 0.01) enriched in aberrant gene expression profiles determined by RNAseq of AML‐MSC in comparison to control HC‐MSC.

In addition, RNA‐seq analysis also revealed gene expression changes in cellular carbohydrate metabolic process, such as glucose metabolic process (GO:0006006), regulation of glucose metabolic process (GO:0010906), and negative regulation of phosphate metabolic process (GO:0045936) (Figure S6, Supporting Information). As shown in **Figure** **4**A, a decreased trend was observed in transcriptional expression of glycolysis genes such as *GP1, PGAM1, ALDOA, LDHA/B*, etc., and an increase in TCA genes such as *SDHA/B/C/D, SUCLA2, ACO2*, etc. PDKs and PDPs are critical for routine metabolism maintenance, which functions as vital regulators of cellular homeostasis.^[^ ^17^ ^]^ The observed changes in PDKs and PDPs may lead to increased activity of mitochondrial pyruvate dehydrogenase complex (PDC). Increasingly research indicates that phosphorylation of PDC yields an array of metabolic changes designed to support anabolism during times of cellular stress and stem cell differentiation.^[^ ^18^ ^]^ Of note, we found that most pentose phosphate pathway (PPP) genes were increased in AML‐cocultured MSCs. PPP generates reducing molecules NADPH, which has been described to modulate stem cell differentiation.^[^ ^19^ ^]^ GO and KEGG analyses were performed to decipher further the differential expressed metabolic genes' biological functions. The results indicated that they were significantly enriched in monosaccharides, glucose, hexose, and pyruvate metabolic processes (Figure 4B). The most significant KEGG pathways of the aberrantly expressed metabolic genes were associated with glycolysis, biosynthesis of amino acids, and citrate cycle (Figure 4C).

Together, these results indicated that OCI/AML3‐cocultured hMSCs had already deviated from the control group of cells at the molecular level. Moreover, expression patterns of representative differentially expressed genes (DEGs) related to lineage differentiation, extracellular matrix organization, and cellular carbohydrate metabolic process were verified in fresh cultured low passage N‐hMSCs versus AML‐hMSCs (Figure S7, Supporting Information). AML‐hMSCs (P0) showed increased mRNA levels of *IL6, CEBPα, PPARγ, COL10, MMP8*, and *ACO2*, but a reduced level of *SDF1α, BMP4, WNT5A*, and *LDHA2*. The drift of these DEGs in primary AML‐hMSCs was consistent with the alterations observed in AML‐cocultured N‐hMSCs.

### 2.4 Allograft AML Mouse Model Suggested In Vivo Effects of AML on MSC Behavior

**Figure 5:**
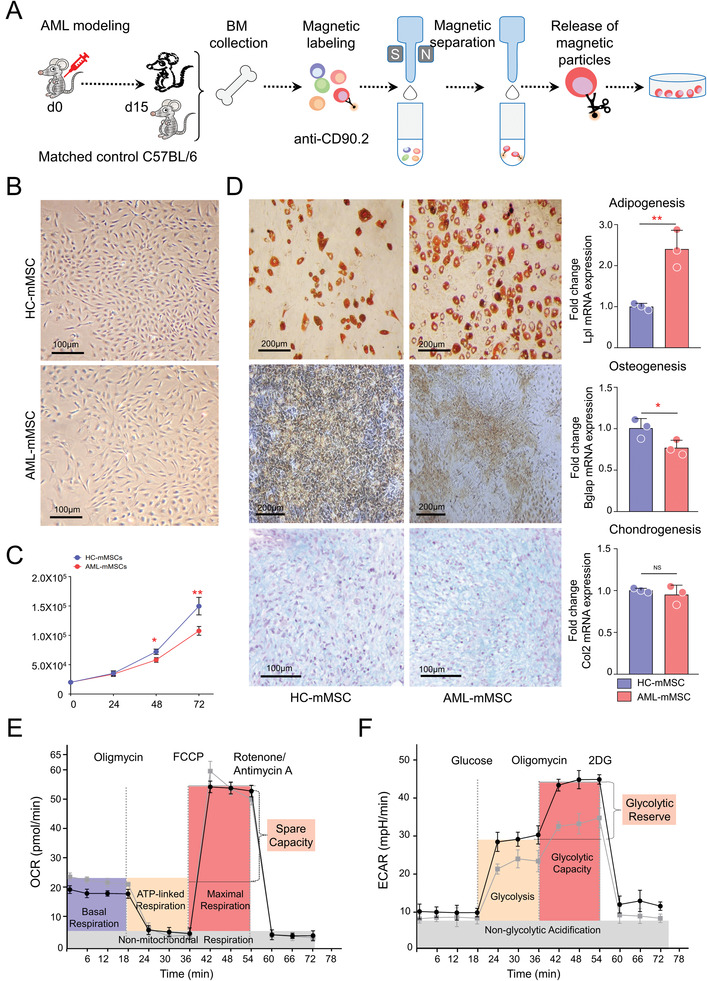
Murine AML cells induced alteration of MSC behavior in vivo. A) Murine AML cell line C1498 was implanted into 12‐weeks‐old C57BL/6 mice by i.v. Injection. Fifteen days later, bone marrow samples were collected from AML mice and age‐matched controls. The bone marrow cells were then labeled with CD90.2 MicroBeads (Miltenyi Biotec 130‐049‐101) to be magnetically retained with the MACS column when placed in the MACS separator. Afterward, the separation column was removed from the magnetic field, and these retained cells were flushed out for RNA extraction or further expansion in plastic dishes. B) Morphology of MSCs (P1) at 85%–90% confluence before subculturing. The enlarged inserts suggested that AML‐mMSCs were flatter and larger than typical spindle‐shaped HC‐mMSCs. Bar: 100 µm. C) Following seeding cells at a concentration of 2 × 10^4^ per 60 mm TC‐treated culture dish, cell counts were determined daily with trypan blue assay. D) Both two types of MSCs differentiated along adipogenic, osteogenic, and chondrogenic lineages with induction of specific medium, including adipocytes (first row), osteoblasts (second row), and chondrocytes (last row). The differentiation degrees were evaluated by qRT‐PCR analysis of lineage marker gene expression. *Gapdh* was used as housekeeping control. The metabolic profile of two MSCs was assessed by measuring the E) OCR and F) ECAR using the Agilent Seahorse XF technology. The black line represents HC‐mMSCs, and the gray line represents AML‐mMSCs. All measures were performed per triplicate. (Unpaired, *n* ≥ 3, two‐tailed Student's, mean with SD, *p < 0.05, **p < 0.01, ***p < 0.001, ****p < 0.0001.)

To further evaluate the in vivo education effects of AML cells on MSCs, an allograft AML mouse model was adopted. As illustrated in **Figure** **5**A, bone marrow samples were flushed out from femurs and tibias of C57BL/6 mice 15 d post‐implantation of murine AML cell line C1498, as well as age‐matched controls. CD90.2^+^ (Thy‐1.2), a glycoprotein present in the MSC membranes, cells were then enriched by MACS Technology using MicroBeads. Alternatively, freshly enriched CD90.2^+^ cells were collected for RNA extraction. We looked into the expression patterns of the above‐mentioned DEGs defined in human samples. As shown in Figure S8A (Supporting Information), AML mice‐derived CD90.2^+^ bone marrow cells exhibited higher mRNA expression levels of adipogenic genes but reduced levels of osteoblastic genes. Changes were also found in cellular metabolic genes. Since mouse MSCs were enriched in the CD90.2^+^ bone marrow cells, these findings hint that MSC behaviors have been changed in the allograft AML mouse models. Finally, CD90.2^+^ plastic‐adherent cells were expanded and passaged, named as healthy control mice‐derived MSCs (HC‐mMSCs) and AML mice‐derived MSCs (AML‐mMSCs) in the present study.

As shown in Figure 5B, MSCs from both groups of mice developed whirlpool‐like monolayers when they reached confluence; meanwhile, morphological variants were found that AML‐mMSCs exhibit a variety of irregular shapes that were much flatter and larger than typical spindle‐shaped HC‐mMSCs. Moreover, AML‐mMSCs showed a reduced proliferative capacity compared to HC‐mMSCs (Figure 5C, *p* < 0.01). Cell surface phenotype analysis by flow cytometry revealed that both types of MSC preparations were positive for CD44, c‐KIT, SCA‐1, CD105, and PDGF, and negative with CD45 and F4/80 (Figure S8B, Supporting Information). Combined with the demonstration of tri‐lineage differentiation potential, an example shown in Figure 5D, we demonstrated that both MSC lines meet the minimum criteria, despite the differences in differentiation efficiency. The histochemical staining results and qRT‐PCR data of lineage‐specific markers indicated that AML‐mMSCs exhibit an increased ability to differentiate into adipocytes but reduced ability in osteoblastic differentiation. There was no significant difference regarding the chondrogenic differentiation between the two groups of cells. Then, the metabolic profile of two MSCs was evaluated by measuring the oxygen consumption rate (OCR, Figure 5E) and extracellular acidification rate (ECAR, Figure 5F). The results suggested that metabolic flexibility was discrepant between the two types of MSCs. AML‐mMSCs exhibited enhanced oxygen consumption and reduced ECAR (Figure 5E,F), facilitating adipogenesis upon induction. Additionally, four metabolites (glucose, lactate, glutamate, and glutamine) were measured and compared between the two types of MSCs using bioluminescent methods from Promega. AML‐mMSCs exhibited increased glucose uptake and glutamine consumption but reduced lactate production compared to HC‐mMSCs (Figure S8C, Supporting Information). Altogether, these data suggest that in vivo AML engraftment induced alternation of MSC behavior such as differentiation potentials and metabolism activity.

### 2.5 Exosome Could be One of the Critical Mediators of AML Cells‐Mediated MSCs Education

**Figure 6:**
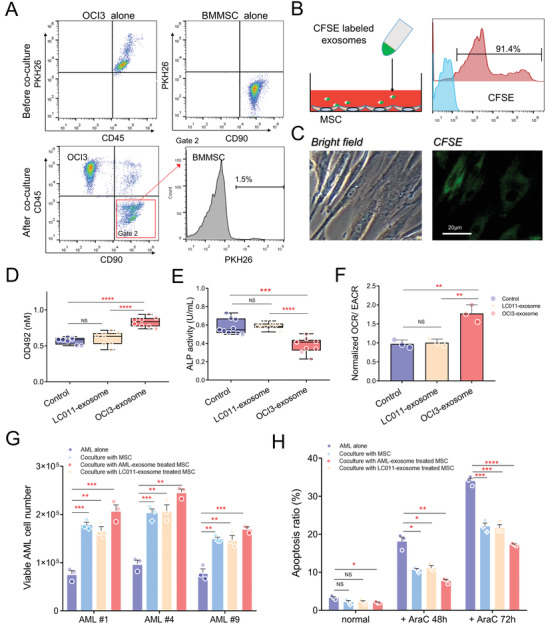
The exosome is a crucial mediator of AML cells‐mediated MSC education. A) OCI/AML3 cells were cocultured with N‐hMSCs for 12 h; then, all cells were harvested and stained for anti‐human CD90 and CD45 flow antibodies. 1.5% of the N‐hMSCs (Gate2: CD45^−^CD90^+^) became positive in the PKH26 dye channel, suggesting material transfer from AML cells to N‐hMSCs. B,C) Exosomes were isolated from cultured OCI/AML3 cells, labeled with fluorescent dye CFSE, and added to the N‐hMSCs culture. Twenty‐four hours later, internalization of the labeled exosomes by N‐hMSCs was confirmed using flow analysis and fluorescence imaging. D,E) N‐hMSCs pre‐treated with AML‐exosome or LC011‐exosome were induced for lineage differentiation; the differentiations were determined as above‐mentioned using Oil Red O staining or ALP staining (*n* = 8). F) Seahorse XF cell energy phenotype tests were performed, and the OCR/ECAR ratio was calculated (*n* = 3). G) Fresh blasts from AML‐M4 patients were cocultured with N‐hMSCs that were pretreated with or without exosomes. Viable cell numbers were determined by cell counter using trypan‐blue 72 h later. H) OCI/AML3 cells cocultured with N‐hMSCs were exposed to 200 × 10^−9^ m AraC, and apoptosis ratios (percent of Annexin V^+^ cells) were determined using flow cytometry. (Unpaired, two‐tailed Student's, mean with SD, *p < 0.05, **p < 0.01, ***p < 0.001, ****p < 0.0001.)

To further study how AML cells influence the behavior of MSCs, we measured the levels of exosomes, which are increasingly recognized as professional carriers of information in cell‐to‐cell communication. To test our hypothesis, OCI/AML3 cells were pre‐stained with PKH26 dye and then directly seeded onto N‐hMSC monolayer at a ratio of 10:1. As short as 12 h of coculture, flow cytometry results showed that ≈1.5% of the N‐hMSCs population became positive in the PKH26 dye channel (**Figure** **6**A), indicating materials were transferring from OCI/AML3 cells to N‐hMSCs. Next, we isolated exosomes from cultured OCI/AML3, labeled with another fluorescent dye CFSE, and incubated them with N‐hMSCs for 24 h. Both flow analysis and fluorescence imaging confirmed that internalized exosomes were found in the majority (>90%) of N‐hMSCs (Figure 6B,C).

Next, we asked whether the uptake of AML‐derived exosomes by N‐hMSCs is responsible for the altered MSC behavior. The effects of AML‐exosome on N‐hMSCs were confirmed, including increased adipogenesis ability (Figure 6D), decreased osteoblastic differentiation capacity (Figure 6E), enhanced oxygen consumption, and reduced ECAR (Figure 6F), which were not found in the group of cells treated with LC011‐derived exosome. In addition, these N‐hMSCs pre‐treated with AML‐derived exosomes showed enhanced supportability to both primary AML blasts (Figure 6G) and leukemia cell lines (Figure 6H) in vitro. Furthermore, a lentiviral shRNA construct was adopted to knock down Rab27a in OCI/AML3 cells, a protein implicated in the exosome release process. The protein level of Rab27a in OCI/AML3‐Rab27a‐shRNA cells (OCI3‐KD) was ≈0.38‐fold relative to specific scrambled control cells (referred to herein as OCI3‐NS) (Figure S9A, Supporting Information). There were no significant changes in cell growth of the transduced cells (Figure S9B, Supporting Information). As expected, exosome secretion was significantly reduced in OCI3‐Rab27a‐shRNA cells compared with controls (Figure S9C, Supporting Information). N‐hMSCs were cocultured with two types of OCI/AML3 cells in the Transwell system for 10 d before being trypsinized and collected for further analysis. Western blotting analyses indicated an upregulation of pAKT in AML‐cocultured N‐hMSCs compared with monoculture control, and the activation level was decreased in the OCI3‐KD‐cocultured group (Figure S9D, Supporting Information). Representative DEGs expression analyses were conducted by qRT‐PCR, and the results were consistent with our previous findings in RNA sequencing array: mRNA levels of genes such as *IL6, GDF15, CEBPα, PPARγ, COL10, MMP8*, and *ACO2* were upregulated in N‐hMSCs cocultured with OCI3‐NS cells compared with monoculture controls, while expression levels of *SDF1α, BMP4, WNT5A*, and *LDHA2* were downregulated. Although N‐hMSCs cocultured with OCI3‐KD cells showed a similar trend in regulating the expression of these genes, the degrees were significantly diminished (Figure S9E, Supporting Information). Similar effects were found in both cell energy tests (Figure S9F, Supporting Information) and adipogenic differentiation assays (Figure S9G, Supporting Information). These results indicated that the phenotypic changes in AML‐educated MSCs were partially reversed due to the reduction of exosome secretion mediated by Rab27a‐shRNA in AML cells.

### 2.6 Proteins Are Differentially Expressed in AML‐Derived Exosomes

**Figure 7:**
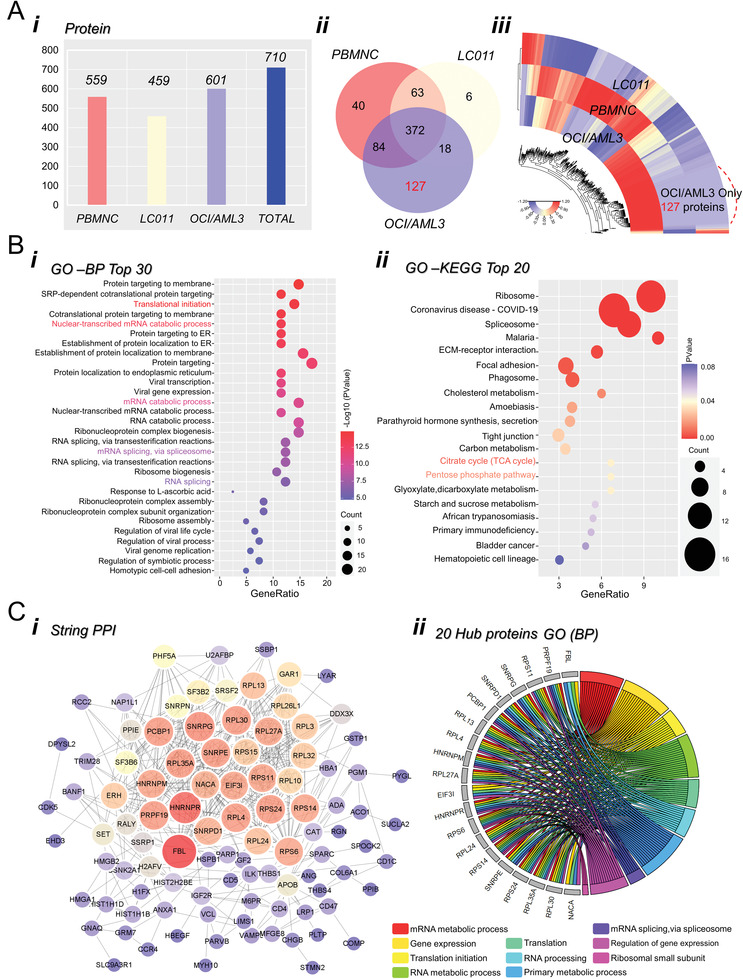
AML‐derived exosomes showed a significant differential abundance of proteins. A) TMT‐based quantitative MS experiments to determine the protein abundances in three groups of exosomes. (i) Number of proteins identified in each group. (ii) The Venn diagram shows the overlap of identified protein numbers among three groups. (iii) Heatmap shows exosomal protein expression profiles. B) Gene ontology annotations (i) and KEGG analysis (ii) of the 127 OCI/AML3 specific exosomal proteins for biological process. C) (i) Protein interaction network of 127 OCI/AML3 specific exosomal proteins. (ii) The top GO enrichment and KEGG analysis of the 20 hub proteins selected from the PPI network using the maximal clique centrality algorithm and the cytoHubba plugin.

Next, we compared protein abundances in exosomes purified from the culture supernatants of OCI/AML3 cells, healthy donor‐derived PBMNCs (peripheral blood‐derived mononucleated cells), and immortal lymphocyte cell line LC011 in a high‐throughput manner using TMT‐based quantitative MS experiments. The raw spectral data were interpreted using Proteome Discoverer 1.4. Eventually, a total of 710 proteins were identified in all three groups of samples (**Figure** **7**A). Among these, 601 proteins were identified in OCI/AML3‐derived exosomes, and the number of proteins identified in PBMNC‐derive exosomes and LC011‐derive exosomes was 559 and 459, respectively. The overlap of identified proteins from different samples was depicted on the Venn diagram. We found that 456 proteins were both expressed in AML‐derived exosomes and PBMNC‐derived exosomes, among which 238 were upregulated (OCI/AML3 > PBMNC) and 218 were downregulated (OCI/AML3 < PBMNC). 390 proteins existed in both AML‐derived exosomes and LC011‐derived exosomes, among which 371 were upregulated (OCI/AML3 > LC011) and 19 were downregulated (OCI/AML3 < LC011). Of note, 127 proteins were identified and quantified only in AML‐derived exosomes. Translational initiation (17/127), proteins composing metabolic process (64/127), biological regulation (61/127), RNA binding (48/127), extracellular membrane (54/127), and vesicle (69/127) were overrepresented. Enriched GO terms and KEGG pathways for the 127 specific expressed proteins were identified. The top 30 enriched GO terms and KEGG pathways are shown in Figure 7B,C; Figure S10 and Table S2 (Supporting Information). The results suggested that the most significantly enriched GO terms were focused on the RNA process. Further, we conducted a PPI network analysis of the 127 proteins using the STRING database (Figure 7Ci). The top 20 hub genes were screened according to their MCC values, including *SNRPD1, SNRPG, SNRPE, HNRNRM, RPS14, RPL35A, RPL30, RPS11, PCBP1, NACA, RPL27A, RPL13, RPL24, RPL4, RPS6, EIF3I, PRPF19, HNRNPR, PRPF19*, and *FBL*. According to the results of the STRING database, we further constructed the PPI network of this top 20 hub genes with a higher degree of connectivity (Figure 7Cii). Based on the GO function analysis, we found that these hub genes were enriched in Translational initiation, Translation, RNA processing, Regulation of gene expression, and Primary metabolic process.

### 2.7 The Pre‐Condition of AML‐Derived Exosomes Facilitate the Leukemia Engraftment and Growth In Vivo

**Figure 8:**
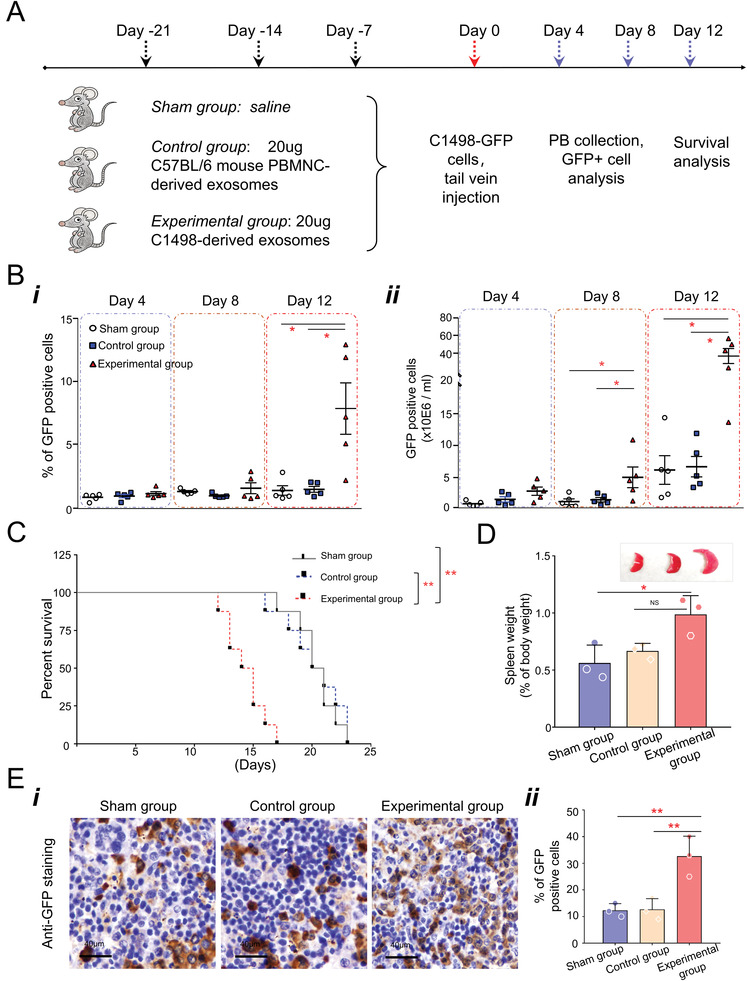
Pretreatment of AML‐derived exosomes facilitated allogeneic leukemia cell growth in mice. A) Schema of experiment design: C57BL/6 mice were randomly divided into three groups (*n* = 11), including sham group, control group, and experimental group; 20 µg of exosomes, harvested from either in vitro cultured C1498 cells of healthy mice‐derived peripheral blood mononuclear cells, were diluted in 10 µl saline and administrated to the mice in individual groups via intrabone injection method once a week for three weeks; mice in the sham group received the same volume of saline for three times; 2 × 10^6^ C1498‐GFP cells were then transplanted into the mice of all three groups via tail vein injection by day 0. Blank arrows: exosome pretreatments; red arrow: C1498‐GFP cell implantation; blue arrows: peripheral blood collection for analysis. B) Blood samples were collected from mouse orbital sinus, and ACK lysis buffer was used to get rid of red blood cells. Then, the percentage of GFP^+^ cells (C1498‐GFP cells) was determined by flow cytometry (i), and the absolute cell counts were calculated (ii). C) Overall survival rate in each group was estimated (*n* = 8). D) Three representative mice per group were sacrificed on day 14. E) Spleens of the AML‐derived exosomes pretreated group were significantly larger than the other groups, and immunohistochemical staining with anti‐GFP antibody (original magnification 40×) confirmed a lower leukemia burden. (One‐way ANOVA, mean with SD, *p < 0.05, **p < 0.01, ***p < 0.001, ****p < 0.0001.)

Given that the uptake of these AML‐derived exosomes induces phenotypic variants in MSCs, which in turn enhanced supportability to leukemia cells in vitro, we hypothesized that the remodeling of the stroma microenvironment contributes to leukemia engraftment and development in vivo. A mouse study was conducted to investigate this hypothesis, as illustrated in **Figure** **8**A. Briefly, C57BL/6 mice were pretreated with either AML‐derived exosomes or mouse peripheral blood mononuclear cells (PBMNC)‐derived exosomes and then transplanted with C1498‐GFP mouse AML cells to monitor the leukemia engraftment. As shown in Figure 8Bi, these mice pre‐conditioned by AML‐derived exosomes showed a significantly higher rate of leukemia cell engraftment by day 12, compared with the other two groups (percentage of C1498‐GFP cells in PB: 3.17% vs 1.10% or 1.08%, all *p* < 0.05). When we looked into the absolute numbers of GFP^+^ cells, significant differences were observed on days 8 and 12 (Figure 8Bii). As expected, the median survival was significantly shorter in mice of the experimental group versus either control groups or sham group (14.5 d vs 20.5 d in both controls; all *p* < 0.01; Figure 8C). Moreover, representative mice were sacrificed on day 14, and the spleens were collected, weighted, fixed, and sliced for IHC staining with an anti‐GFP antibody. As shown in Figure 8D,E, the analysis further confirmed a higher leukemia burden in mice pre‐conditioned by AML‐derived exosomes. Taken together, these data suggested that the pre‐condition of AML‐derived exosomes facilitates leukemia growth in vivo.

## 3 Discussion

MSCs and their lineage differentiated cells, such as osteoblastic cells and adipogenic cells, are essential components of the bone marrow hematopoietic microenvironment and play critical roles to modulate hematopoiesis through both cell‐cell interactions and paracrine secretions.^[^ ^2^, ^20^ ^]^ In recent years, it is becoming increasingly clear that leukemia cells induce various stromal alterations in the bone marrow niche and hijack the homeostatic mechanisms of normal HSC to support leukemic progression. Yet, the mechanisms remain primarily undefined.^[^ ^21^ ^]^ Our group and others have found that AML patient‐derived MSCs are phenotypically and functionally different from normal MSCs with distinct genotypes and protein expression.^[^ ^8^, ^22^ ^]^ The educated MSCs were proposed to be pivotal for the development of AML chemoresistance, thus contributing to disease relapse.

The present study showed that AML cells educate MSCs to use OXPHOS more than glycolysis during energy production. Therefore, observing increased oxygen consumption and respiratory enzyme complex activation in AML‐MSCs becomes logical. Studies have proved that quiescent MSCs maintain a relatively low level of mitochondria activity and can develop into an activated stage once upon induction of differentiation.^[^ ^16^, ^23^ ^]^ Indeed, increasing shreds of evidence suggested that the differentiation capability of leukemia patient‐derived MSCs is altered, which might represent specific mechanisms of leukemogenesis.^[^ ^12^, ^13^, ^14^, ^15^ ^]^ However, the results were controversial, as some researchers reported that these leukemia cell‐educated MSCs tend to differentiate into osteoblastic cells.^[^ ^15^ ^]^ In contrast, others suggested they were easier to turn into adipocytes.^[^ ^13^, ^14^ ^]^ The inconsistent results are not surprising due to the complexity of leukemia diseases. Different research groups obtained MSCs from different leukemia subtypes at various stages may lead to variant results. We focused on the AML subtype of AML‐M4 (FAB‐M4) in the current study. Still, considerable variations were observed in the differentiation capability of AML‐hMSCs, as well as AML blasts' influence on N‐hMSC differentiation.

Nevertheless, our study found that both AML‐MSCs and AML cells‐cocultured normal MSCs (both refer to AML cells‐educated MSCs) exhibit an increased ability to differentiate into adipocytes and reduced ability in osteoblastic differentiation. Consistent with our observation, changes were observed in genes associated with lineage differentiation in AML‐cocultured MSCs. Analysis of upregulated genes indicated that the enriched GO terms involved adipogenic differentiation, such as fat cell differentiation (GO:0045444) and regulation of fat cell differentiation (GO:0045598); on the other hand, enriched GO terms including regulation of osteoblast differentiation (GO:0045667), regulation of bone mineralization (GO:0030500), and positive regulation of bone mineralization (GO:0030501) were observed in the downregulated genes (Figure S5, Supporting Information). Indeed, bone marrow adipocytes were reported to support the survival and proliferation of AML blasts, which induce free fatty acid release via activation of lipolysis.^[^ ^24^ ^]^ Moreover, leukemic cells were presented with high fatty acid oxidation (FAO) rate, indicating that these cells benefit from the lipolysis. Thus, the enhanced adipogenesis of MSCs might represent one of the mechanisms of increased engraftment of C1498 leukemic cells in mice.

ECM is collagen and elastic fibers composite embedded in a viscoelastic gel of proteoglycans, hyaluronan, and various glycoproteins. The ECM provides a 3D structural and biochemical support and functions as one of the most critical microenvironmental parameters that regulate the cell fates of both MSCs and leukemia cells. Of note, a significant enrichment of extracellular matrix (GO:0031012) was evident among differential expression genes in AML cells‐educated MSCs: Matrix metalloproteinases 1 (MMP1), MMP3, and MMP8 were upregulated 12.3‐, 16.9‐, and 7.3‐fold in AML‐cocultured MSCs, respectively. The expression of different types of collagenase showed various changes, COL5A3, COL6A3, COL7A1, and COL10A were upregulated, while COL8A1, COL11A1, COL12A1, COL14A1, and COL25A1 were downregulated (Figure S6, Supporting Information). These results suggest active extracellular matrix (ECM) turnover in the leukemic microenvironment that favors adipogenic differentiation and leukemia engraftment. Further studies of the mutual interactions among ECM remodeling, MSC fate decision, and leukemia progression will contribute to the development of new therapeutics.

Exosomes are biological active membrane‐bound extracellular vesicles that consist of proteins, mRNA, and noncoding miRNA.^[^ ^25^ ^]^ In recent years, the role of exosomes as mediators between cancer cells and tumor microenvironment has gained increasing attention.^[^ ^26^ ^]^ For example, stressed MSC‐derived exosomes have been shown to promote or inhibit cancer cell proliferation and metastasis via transferring tumor‐supportive miRNAs and proteins;^[^ ^27^ ^]^ in turn, breast cancer‐derived exosomes were reported to suppress glucose uptake by stromal cells and fibroblasts;^[^ ^28^ ^]^ exosomes released from CML cells stimulated MSCs to produce IL 8;^[^ ^10^ ^]^ AML‐derived exosomes induced a broad downregulation of HSC‐supporting factors (e.g., CXCL12, KITL, and IGF1) in MSCs and reduced their ability to support normal hematopoiesis.^[^ ^21^, ^29^ ^]^ Our findings suggested that the internalized AML cells‐derived exosomes are at least partially responsible for AML‐induced MSCs metabolism alteration and cell fate decision. Furthermore, intrabone administration of AML cells‐derived exosomes remodeled the bone marrow microenvironment into a leukemia‐favorable state. Despite clear evidence for the existence of these interactions, the precise repercussions on the reprogramming of MSCs are still poorly understood.

So far, the majority of the exosome studies in cancer biology have focused on the exosomal noncoding RNAs, which have shown significant functions in regulating cancer progression.^[^ ^30^ ^]^ In contrast, the protein signatures in exosomes were relatively less studied. It was reported that the protein composition in total exosome fractions isolated from peripheral blood is significantly higher in cancer patients than in healthy individuals. For example, the protein concentration in serum‐derived exosomes from AML patients was 60‐fold higher than those isolated from the serum of normal controls.^[^ ^31^ ^]^ Recent works also proved particular regulated proteins in exosomes of malignant cells.^[^ ^32^ ^]^ Here, we present a systematic protein analysis of exosomes and show that AML‐derived exosomes exhibited significantly different protein expression patterns compared to normal hematopoietic cells. Interestingly, we found that some of the highly loaded proteins in AML‐derived exosomes also had been reported to be generally higher expressed in AML‐MSCs,^[^ ^8c^ ^]^ including PPP2R1A, CDK4, PARP1, NOTCH1, and CSNK2A1; furthermore, we found these proteins were also highly enriched in AML‐patient serum‐derived exosomes in preliminary experiments with a limited number of samples (*data not shown*), suggesting that this group of proteins may of great values in AML early detection and diagnosis.

Evolving evidence suggests that the bone marrow microenvironment function as a crucial factor in leukemogenesis, progression, and chemoresistance in a disease‐specific manner. The present study shows that AML cells transfer functional materials via exosomes to educate MSCs toward a leukemia‐permissive phenotype and contribute to leukemia progression. Further studies of the characterization of cargo molecules in AML‐derived exosomes will better develop our understanding of the origins of disease and provide the potential for new avenues of therapy.

## 4 Experimental Section

### Cell Lines and Cultures

The study protocol (No. IIT1222) was approved by the Institutional Review Board of Zhejiang University and was performed following the guidelines and regulations established by Zhejiang University to protect human subjects. Human OCI/AML3 and mouse AML cell line C1498 were purchased from the American Type Culture Collection (ATCC); immortalized lymphocyte cell line LC011 was established by in vitro infecting human peripheral blood lymphocytes (55‐year‐old male healthy volunteer without diabetes, blood diseases, or tumors) with Epstein Barr Virus (EBV). Rab27a small hairpin RNA (shRNA) and nonsilencing (NS) shRNA control were purchased from WZ Biosciences Inc. for lentiviral construct. C1498‐GFP cells were engineered by retroviral transduction using the pLEGFP plasmid (Addgene). GFP expression by C1498‐GFP was maintained with G418 and monitored by flow cytometry. The above‐mentioned cell lines were cultured in RPMI 1640 medium (Invitrogen) supplemented with 10% fetal bovine serum (FBS, Gemini).

### Isolation, Culture, and Lineage Differentiation of Mesenchymal Stromal Cells

MSCs were isolated and purified through their physical adherence to the plastic cell culture plate as previously described.^[^ ^4^, ^33^ ^]^ Cells were harvested before reaching confluence by applying 0.25% trypsin and 1 × 10^−3^ m EDTA (Invitrogen). Then, MSCs were checked for positivity of CD105, CD73, and CD90 and the lack of expression of CD45 and CD34 using flow cytometry.

For mesenchymal lineage differentiation, MSCs were grown to 95% confluence in MEM‐*α* medium containing 10% FBS and then changed to specific differentiation medium (Osteo‐Diff medium, Adipo‐Diff medium, and Chodron‐Diff medium, Miltenyi Biotec). Osteogenic differentiation was shown by staining for calcium deposition, adipogenic differentiation with Oil Red, and chondrogenic differentiation with Alcian blue. RNAs were extracted from MSCs before and after differentiation, and Real‐time RT‐PCR was performed to determine the expression of lineage‐related genes.

### Co‐Culture of MSCs and AML Cells with Transwell System

MSCs were seeded at 2 × 10^3^ cells/cm^2^ in the lower chamber of 75 mm Transwell (0.4 µm Pore Polycarbonate Membrane Insert, Corning) for 12 h before the addition of AML cells. Then, 5 × 10^3^ OCI/AML3 cells were loaded into the upper chamber. The exact amount of LC011 was loaded in the control group instead of OCI/AML3 cells. The medium was changed twice a week, and the coculture system was maintained for 9–10 d until hMSCs reached confluence.

### Western Blot Analysis

Boiled lysates (in Laemmli buffer) were separated on 10% polyacrylamide gels, and proteins were transferred onto nitrocellulose membranes for immunoblotted with primary antibodies. Following subsequent application of secondary antibodies, signals were visualized using an ECL system (CWBIO) and quantified by Image J software. CD81, TSG101, p‐Akt, Akt, and GAPDH antibodies were purchased from Santa Cruz Biotechnology. anti‐Rab27a and anti‐*β*‐ACTIN were from Proteintech.

### Seahorse Metabolic Analysis of Cultured MSCs

Cell metabolism measurements were performed using the XF96 Extracellular Flux analyzer (Seahorse Bioscience), which provides an overview of glucose metabolism through oxygen consumption rate (OCR) and extracellular acidification rate (ECAR) output in response to separate treatments. Oligomycin is used as a complex V inhibitor to assess ATP production levels. FCCP is used as a mitochondrial uncoupler to assess maximal respiration, 2‐DG is used as a glucose analog to inhibit glycolysis, and rotenone treatment is used as a mitochondrial complex I uncoupler to assess non‐mitochondrial respiration. OCR and ECAR were normalized against cell counts.

### Measurement of Multiple Metabolites in Cultured MSCs

Four metabolites, including glucose, lactate, glutamate, and glutamine, were parallelly measured using the bioluminescent Glucose‐GLoTM (Promega #J6021), Lactate‐GloTM (Promega #J5021), and Glutamine/Glutamate‐Glo (Promega #J8021) Assays according to the instructions. Briefly, MSCs were plated at a 5000 cells/well concentration in 100 µL MEM‐*α* with 5 × 10^−3^ m glucose, 2 × 10^−3^ m glutamine, and 10% dialyzed serum. At the indicated time points (8, 24, and 48 h), 2.5 µL of the medium was collected, diluted in 97.5 µL PBS, and stored at −20 °C. At the end of the experiment, samples were thawed, aliquoted, and detected, respectively.

### Transcriptome‐seq and Bioinformatic Analysis

Total RNAs were isolated using TRIzol‐based standard procedure. The purity and quantity of RNAs were estimated by Nano photometer (IMPLEN) and RNA Nano 6000 Assay kit (Agilent Technologies). cDNA libraries were generated using NEB Next Ultra Directional RNA Library Prep Kit (NEB) and sequenced on an Illumina Hiseq TM2000/Miseq. Trimmomatic v0.36 is used for quality filtering to ensure that all sequences were above 36 bp. Paired‐end clean reads were then mapped to the human reference genome (NCBI: Homo sapiens. GRCh38.94.gtf) by using Hisat2. After alignment analysis, each gene's FPKM or TPM value was determined and then analyzed using the R Bioconductor DESeq2 package (the negative binomial distribution). Genes with an adjusted *p*‐value < 0.05 and log_2_ ^(fold change)^ >1 were assigned as differentially expressed. Gene Ontology enrichment (GO) and Kyoto Encyclopedia of Genes and Genomes (KEGG) analysis was performed using the Cluster Profiler R package. The RNA sequencing (RNA‐Seq) data were deposited into Sequence Read Archive (SRA) with the Bio‐project ID of PRJNA749678.

### Exosome Isolation

RPMI 1640 media supplied with 10% FBS was centrifuged at 100 000*g* for 10 h to exclude bovine exosome contamination. Then, the centrifuged media was filtered using a 0.2 µm filter and prepared for cell culture. Supernatants were collected 48 h later and first centrifuged at 800*g* for 10 min at 4 °C. Exosomes were further isolated using standard ultracentrifugation protocol or total exosome isolation reagent (ThermoFisher). The exosome pellets were resuspended in PBS or saline. A fraction of the resuspended exosome was lysed in RIPA buffer and quantified using a BCA protein assay kit for protein quantification.

### Quantitative Proteomic Analysis

Exosome proteomics services were supported by Wayen Biotechnologies following a standard protocol.^[^ ^34^ ^]^ The analyses include tandem mass tag labeling, high‐performance liquid chromatography (HPLC: EASY‐nLC 1000) separation, and mass spectrometry (Orbitrap Elite, Thermo Scientific). Max‐Quant 1.5.8.3 (Max‐Planck Institute for Biochemistry) was used as a quantitative proteomics software package for analyzing large mass spectrometry data sets. Metascape (http://metascape.org) and cluster profiler package o were used for gene annotation and enrichment analysis. Terms with *p*‐value < 0.05, minimum counts of 3, and enrichment factor > 1.5 were collected and grouped into clusters based on their membership similarities.

### Mouse Study

Male C57BL/6 mice, aged ten weeks, were purchased from Shanghai SLAC Laboratory Animal Co. All animal procedures were performed according to protocol (No. 18021) approved by the Institutional Animal Care and Use Committee at Zhejiang University. Mice were randomly divided into three groups and treated with C1498‐derived exosome, regular mouse PBMNC‐derived exosome, or the same volume of PBS, respectively. After the pre‐condition, the mice were then intravenously injected with 2 × 10^6^ cells C1498‐GFP cells. Beginning on day 4 after C1498 implantation and periodically after that, blood was drawn from the retro‐orbital venous plexus of mice and resuspended in ACK lysis buffer to remove red blood cells. The remaining cells were washed twice with PBS and analyzed by flow cytometry for GFP‐expressing cells. The percentage of C1498‐GFP cells in peripheral blood was calculated by gating on the entire white blood cell (WBC) population. The absolute cell counts were determined using counting beads assays (ThermoFisher). Fourteen days after the transplantation of C1498 cells, representative mice were humanely sacrificed by CO_2_ asphyxiation. The extent of leukemic infiltration was assessed by staining GFP^+^ leukemic cells with an anti‐GFP antibody (Abcam).

### Statistical Analysis

All data are presented as means ± standard deviations from at least three independent assays. Statistical analysis between groups was carried out using the unpaired, two‐tailed Student's *t*‐test (or ANOVA) contained in the GraphPad Prism program. The Log‐rank (Mantel‐Cox) test in the GraphPad Prism program was used for the survival curve comparison calculations. Differences were considered significant at a *p* < 0.05.

## Conflict of Interest

The authors declare no conflict of interest.

## Author Contributions

L.Z. and Q.Z. contributed equally to this work. Y.C. and Y.Y. conceptualized and planned the study. L.Z., Q.Z., H.C., Z.W., X.H., and R.P. performed the experiments and analyzed the data. X.H. and R.P. performed gene expression array experiments, and the data were analyzed by L.Z., Y.Y., and Y.C. interpreted the data and wrote the manuscript.

## Supporting information

Supporting Information

## Data Availability

The data that support the findings of this study are available from the corresponding author upon reasonable request.
