# Supplementary material for: Acute Myeloid Leukemia Cells Educate Mesenchymal Stromal Cells toward an Adipogenic Differentiation Propensity with Leukemia Promotion Capabilities
Source: Adv Sci (Weinh). 2022 Mar 20;9(16):2105811. doi: 10.1002/advs.202105811 (PMC9165478; doi:10.1002/advs.202105811)
Supplement: Supplementary file 1 — Supporting Information [file ADVS-9-2105811-s001.pdf]

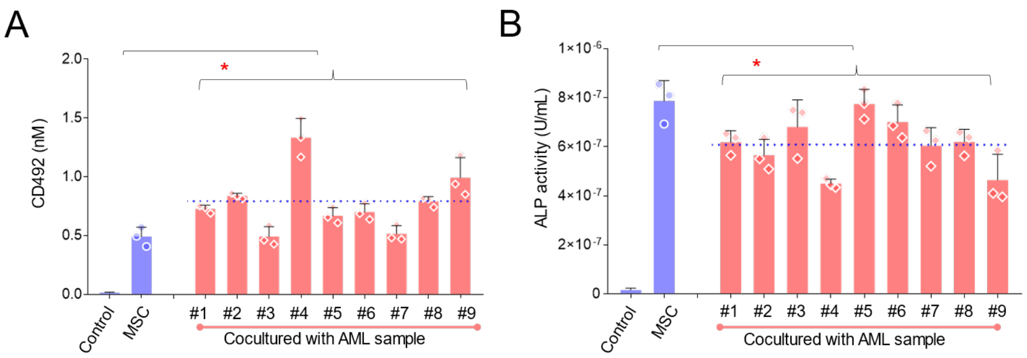

Supplementary Figure 1. Primary AML blasts cultured with N-hMSCs. N-hMSCs at passage 3 were co-cultured with fresh blasts from patients diagnosed as AML-M4. (A) Adipocytic lineage differentiation was determined using Oil Red O staining and determined in a microplate reader at 492 nm after extraction by isopropanol. (B) The osteogenetic potential of MSCs with or without AML blasts incubation was revealed by ALP activity after 21 days differentiated in osteogenesis medium. (Mean ± SD, n≥ 3, *p<0.05, **p<0.01, ***p<0.001, ****p<0.0001).

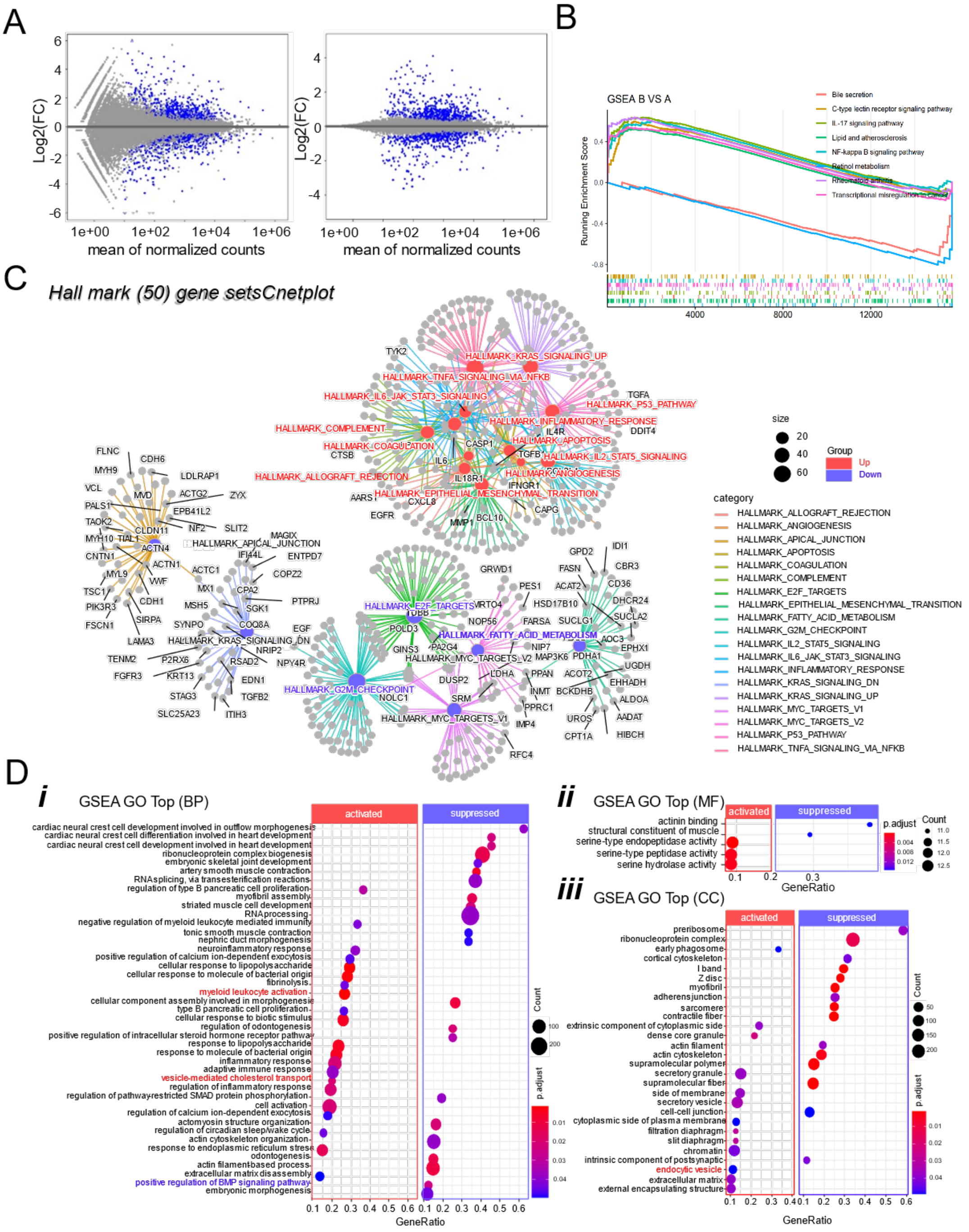

Supplementary Figure 2. GSEA analysis of all detected protein-coding genes. (A) The MA plot shows $\log_2$ base means versus the $\log_2$ fold-change for all detected genes. The original FC data (left panel) was corrected by lfcshrink (right panel) before GSEA analysis. (B) GSEA of eight KEGG pathways with the FDR q values <0.05. (C) Network plot of the screened hallmark gene sets as shown in Figure 3C. (D) The top GO annotations included BP, MF, and CC in the enrichment analysis of the protein-coding genes.

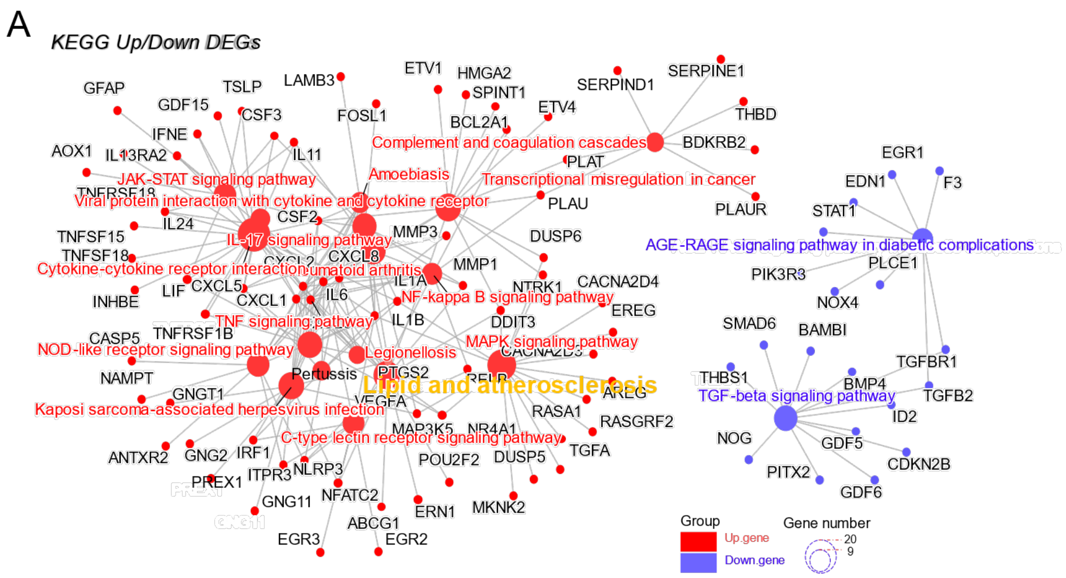

Supplementary Figure 3. Network plot of the most regulated KEGG pathways as shown in Figure 3F

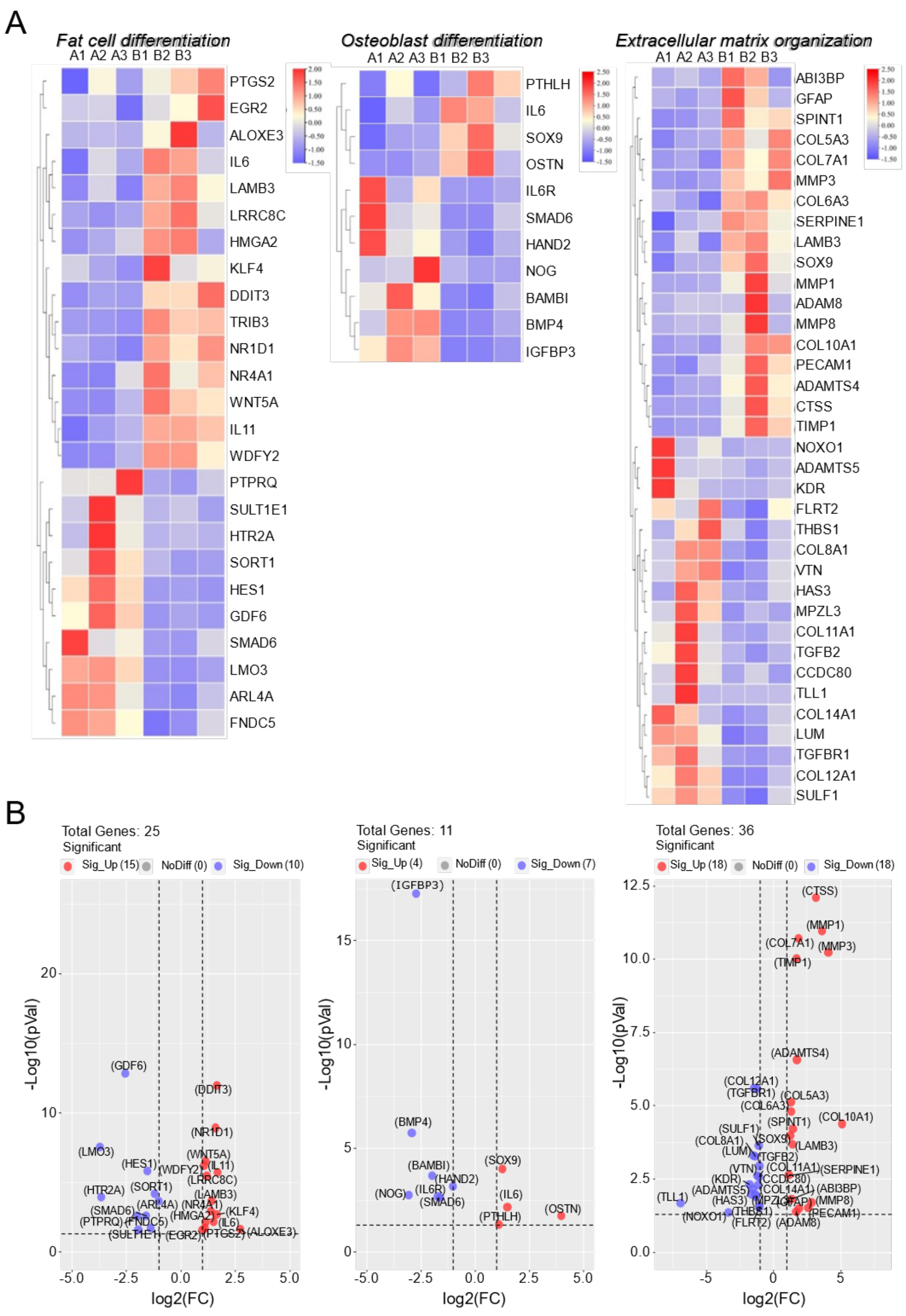

Supplementary Figure 4. Significantly regulated genes in three representative enriched GO terms. (A) Heatmap showing the expression profiles of regulated genes. (B) Volcano plots show regulated genes; red dots represent up-regulated genes, and blue dots represent downregulated genes.

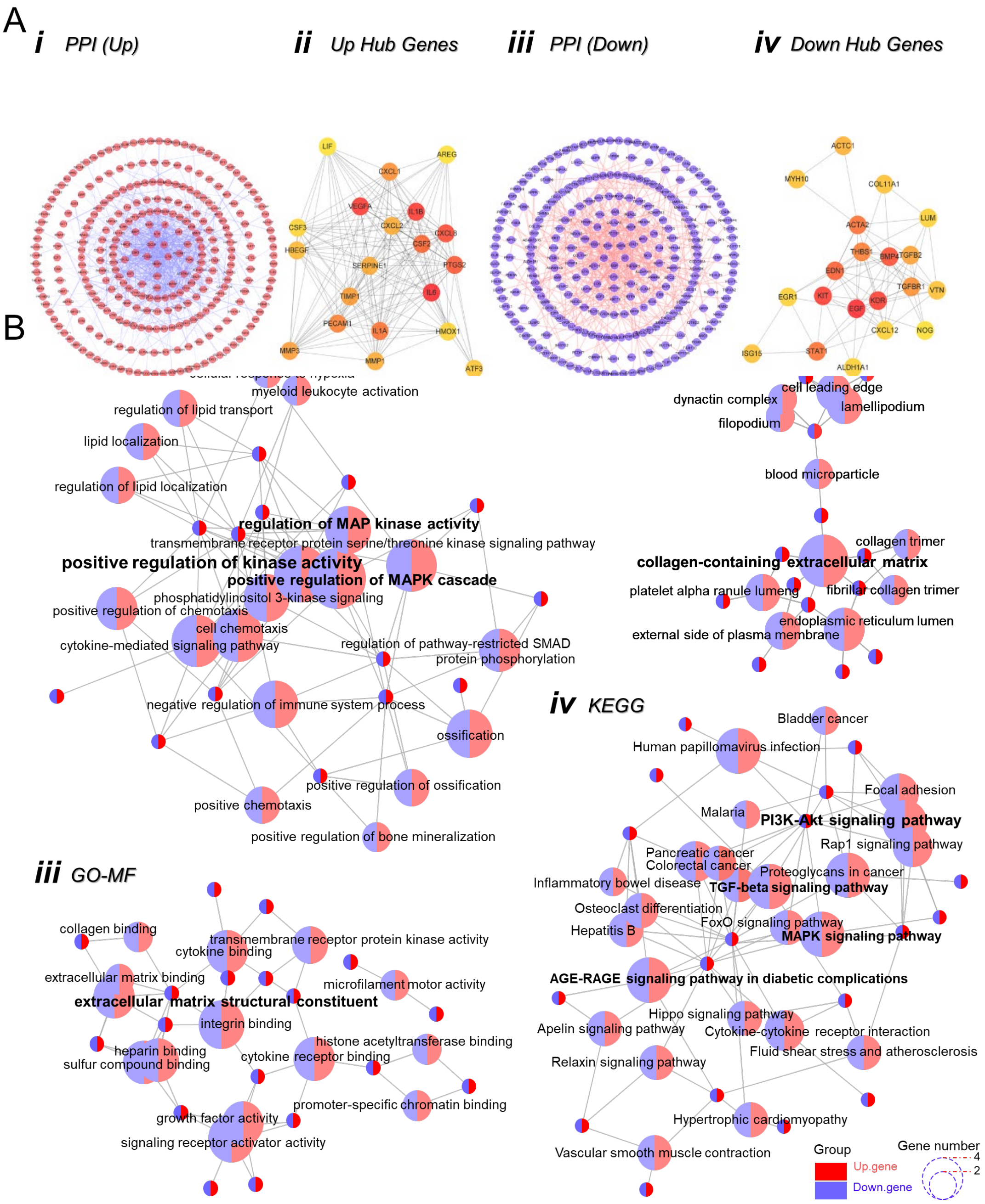

Supplementary Figure 5. Protein-protein interaction (PPI) analysis of the DEGs. (A) PPI network constructed by STRING was visualized with Cytoscape. Up-regulated genes ae shown in red (i), whereas down-regulated ones are blue (iii). The 20 hub proteins selected from the PPI network were visualized using the maximal clique centrality algorithm and the cytoHubba plugin (ii, iv). (B) The top GO enrichment and KEGG analysis of the 40 hub proteins.

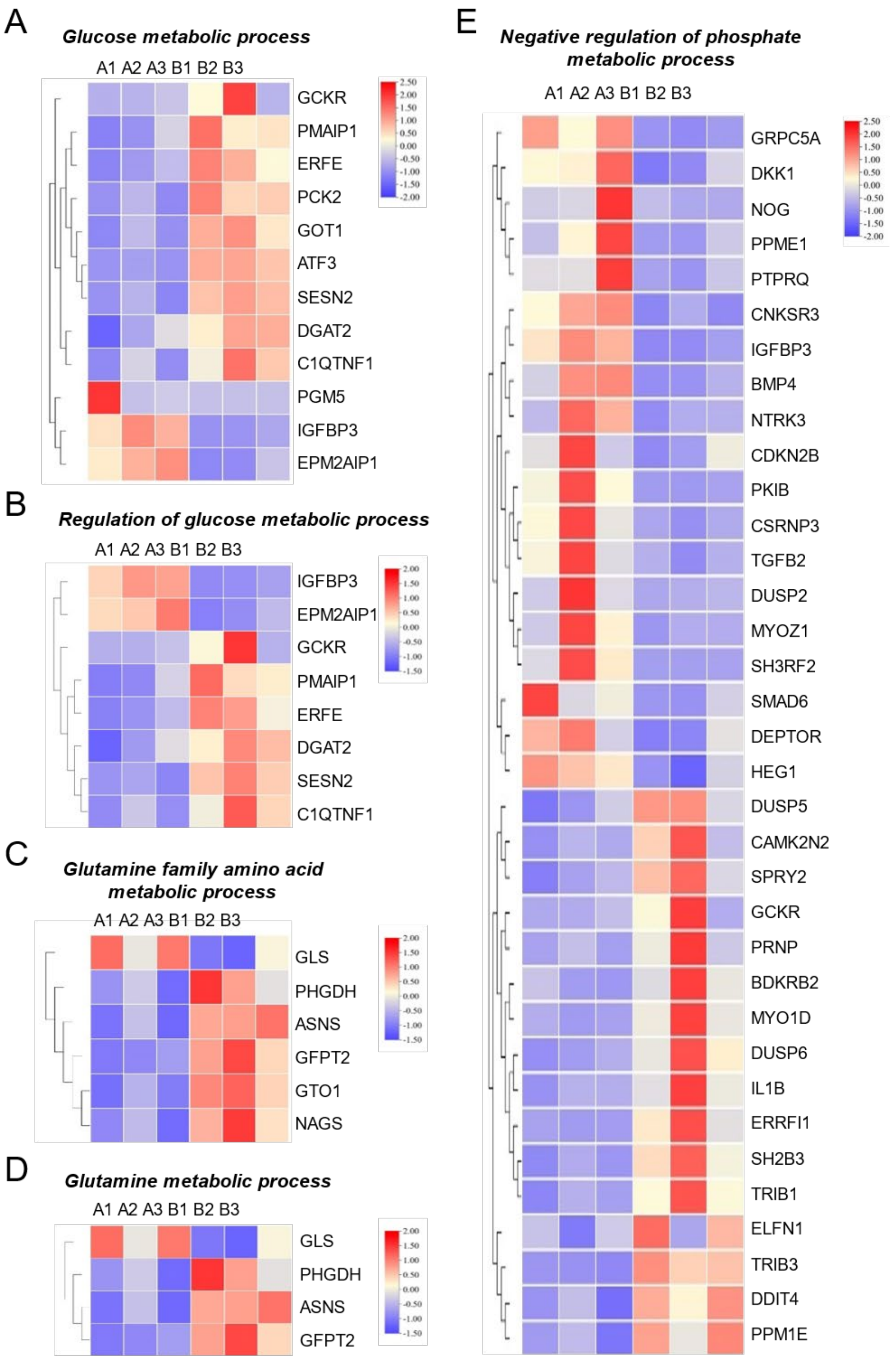

Supplementary Figure 6. Representative enriched GO terms of cellular carbohydrate metabolic process. Heatmap plots showing the expression profiles of differential expressed genes among glucose metabolic process (A), regulation of

glucose metabolic process (B), Glutamine family amino acid metabolic process (C), glutamine metabolic process (D), and negative regulation of phosphate metabolic process (E).

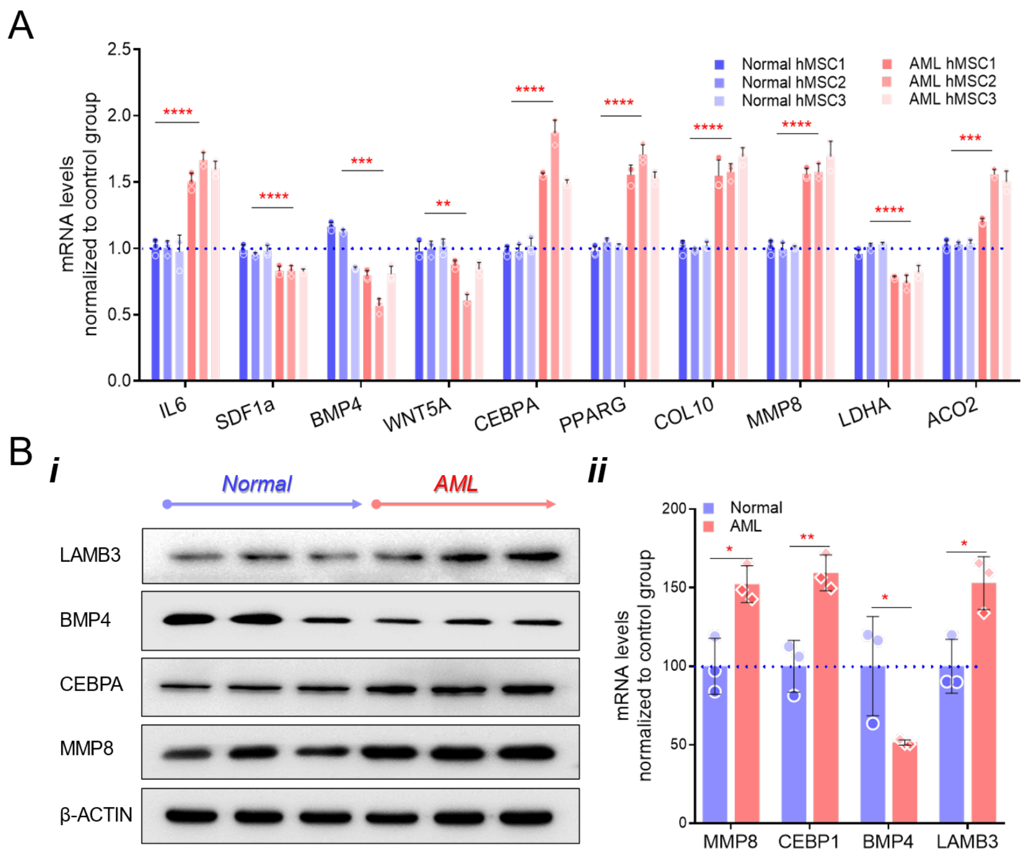

Supplementary Figure 7. Determination of representative EDGs in N-hMSCs and AML-hMSCs. (A) Three AML-hMSCs (P3) and age-matched N-hMSCs (P3) were harvested for RNA extraction. mRNA expression levels of *IL6, CEBPα, PPARγ, COL10, MMP8, ACO2*, *SDF1α, BMP4, WNT5A,* and *LDHA2* were determined using qRT-PCR. (B) Protein levels of LAMB3, BMP4, CEBPA, and MMP were evaluated by Western blot analysis. β-ACTIN was adopted as housekeeping control. (Mean ± SD, n=6, *p<0.05, **p<0.01, ***p<0.001, ****p<0.0001).

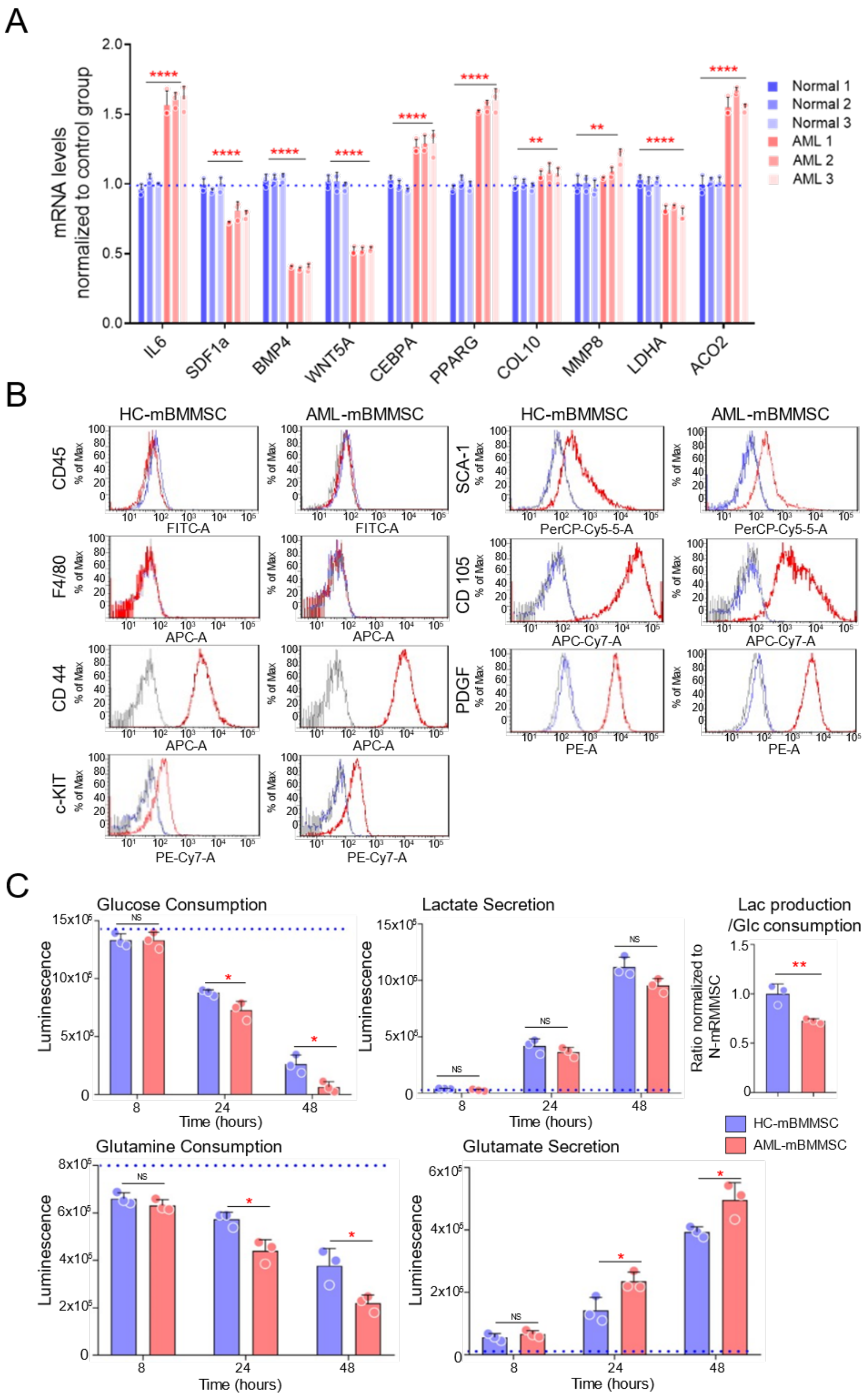

Supplementary Figure 8. Characteristics analysis of mouse MSCs. (A) Bone marrow cells were harvested from AML mice and matched normal controls. CD90.2^{+}

cells were further enriched using MACS and collected for RNA extraction. The expression of representative DEGs was determined by qRT-PCR and compared between two groups of cells. (B) The expanded MSC preparations were checked for positivity of CD44, c-KIT, SCA-1, CD105, and PDGF, and the lack of expression of CD45 and F4/80. (C) Extracellular metabolites were measured. The blue dotted lines depict the signals from control wells containing medium but without cells. All measurements were performed per triplicate. (Mean with SD, *p<0.05, **p<0.01, *** p<0.001, **** p<0.0001).

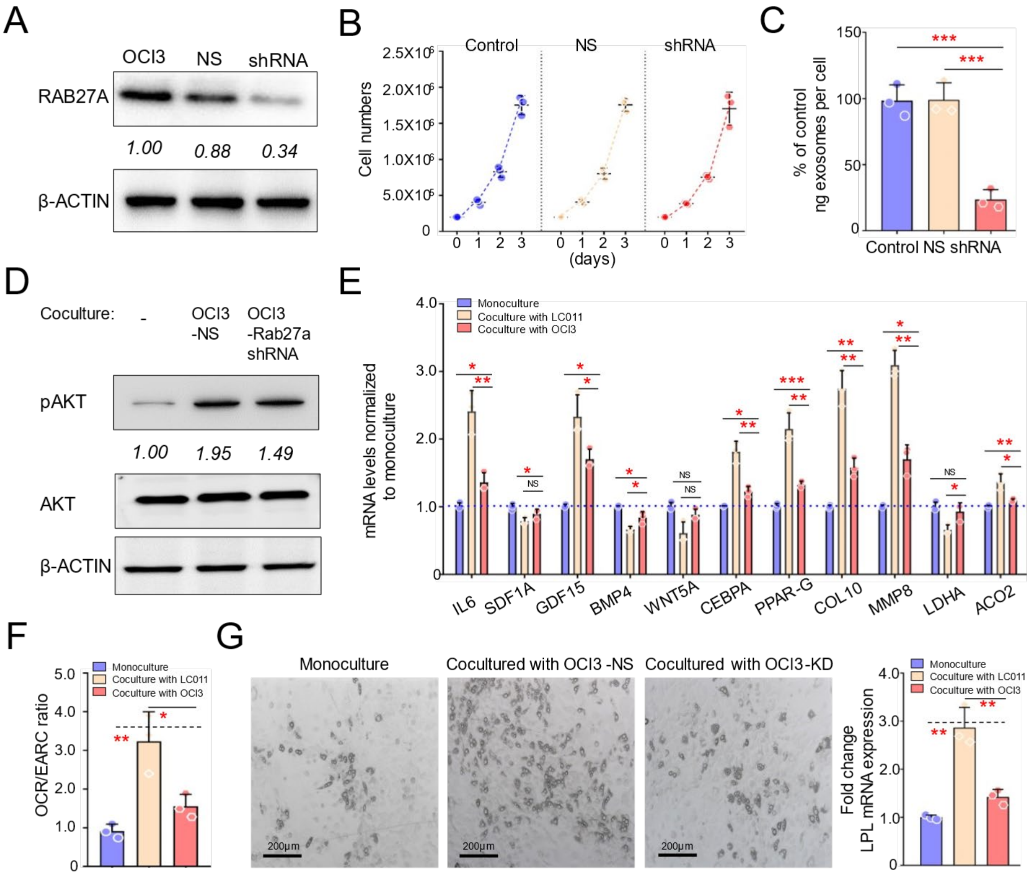

Supplementary Figure 9. The exosome is a key mediator of AML cells-mediated MSC education. (A) Rab27a expression in OCI-AML3 cells was knocked down by shRNA. (B) There was no significant change in the cell growth of the transduced cells. (C) The OCI3-Rab27a-shRNA cells showed a significantly reduced level of exosome production compared with NS controls. (D) Western blotting assays were performed. Activation of pAKT was evaluated and normalized to AKT. (E) Representative gene expression analyses were conducted by qRT-PCR. (F) Seahorse XF cell energy phenotype tests were performed for three types of MSCs, and the OCR/ECAR ratio was calculated. (G) After two weeks of incubation in an adipogenesis induction medium, abundant numbers of adipose drops can be directedly visualized under a phase-contrast microscope. The differentiation degrees were evaluated by qRT-PCR analysis of LPL expression. (one-way ANOVA, mean with SD, * $p<0.05$, ** $p<0.01$, *** $p<0.001$, **** $p<0.0001$)

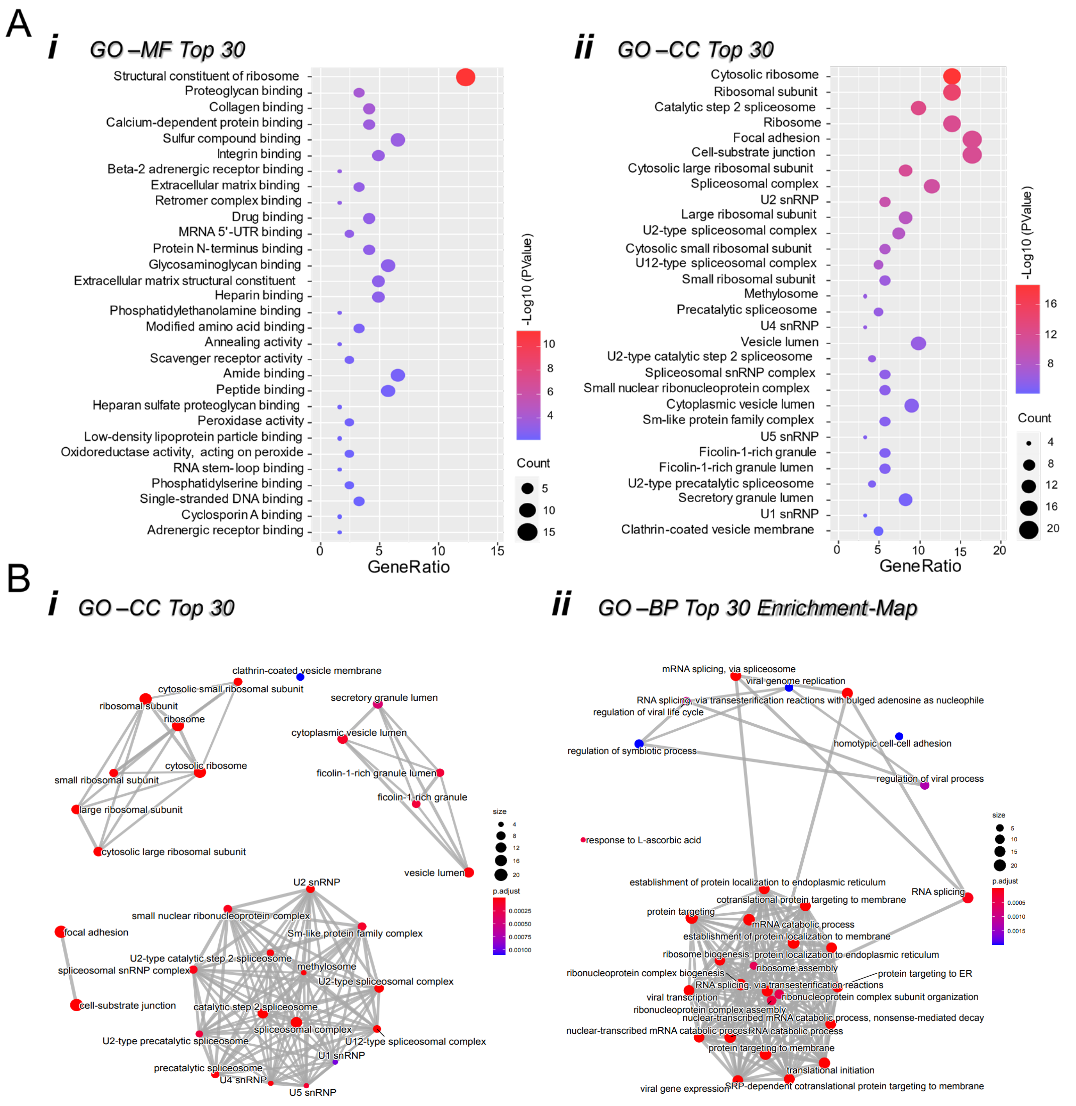

Supplementary Figure 10. Enrichment analysis of KEGG and GO terms for exosomal proteins. (A) Bubble plots of the top 30 GO-MF(i) and GO (CC) terms (ii) of the 127 exosomal proteins that were only identified explicitly in OCI/AML3. (B) Enrichment map of top 30 GO-CC and BP terms.

Supplementary Table 1 Characteristics of AML patients and healthy donors

ID	Age (years)	Gender	WBC (10^9/L)	HGB (g/L)	BM leukemic blasts (%)	FAB Subgroup	Previous treatment
AML1	63	*Female*	80.1	4.4	53.1	M4	*No*
AML 2	57	*Female*	52.3	8.7	62.3	M4	*No*
AML 3	51	*Male*	32.0	9.0	36.7	M4	*No*
AML 4	48	*Male*	83.1	4.7	56.0	M4	*No*
AML 5	65	*Female*	256.3	6.1	90.7	M4	*No*
AML 6	53	*Male*	124.7	8.5	89.9	M4	*No*
AML 7	55	*Female*	56.4	8.3	56.6	M4	*No*
AML 8	57	*Male*	38.6	5.4	64.3	M4	*No*
HD 1	53	*Male*	4.2	14.4			
HD 2	61	*Male*	5.4	15.2			
HD 3	55	*Female*	6.1	12.4			
HD 4	58	*Female*	5.5	11.8			

WBC, white blood cells; HGB, hemoglobin; BM, bone marrow; FAB, AML according to WHO French-American-British classification

Supplementary Table 2 Enriched GO and KEGG pathways results

Description	Count	Type	GeneRatio	pvalue	geneID
protein targeting to membrane	18	BP	18/122	1.8E-15	VPS37B/SDCBP/RPS24/RPS15/RPS14/RPS11/RPS6/RPL35A/RPL32/RPL27A/RPL30/RPL24/RPL13/RPL10/RPL4/RPL3/NACA/CDK5
SRP-dependent cotranslational protein targeting to membrane	14	BP	14/122	5.8E-15	RPS24/RPS15/RPS14/RPS11/RPS6/RPL35A/RPL32/RPL27A/RPL30/RPL24/RPL13/RPL10/RPL4/RPL3
translational initiation	17	BP	17/122	6.7E-15	EIF3I/RPS24/RPS15/RPS14/RPS11/RPS6/RPL35A/RPL32/RPL27A/RPL30/RPL24/RPL13/RPL10/RPL4/RPL3/HSPB1/DDX3X
cotranslational protein targeting to membrane	14	BP	14/122	1E-14	RPS24/RPS15/RPS14/RPS11/RPS6/RPL35A/RPL32/RPL27A/RPL30/RPL24/RPL13/RPL10/RPL4/RPL3
nuclear-transcribed mRNA catabolic process, nonsense-mediated decay	14	BP	14/122	3.9E-14	RPS24/RPS15/RPS14/RPS11/RPS6/RPL35A/RPL32/RPL27A/RPL30/RPL24/RPL13/RPL10/RPL4/RPL3
protein targeting to ER	14	BP	14/122	3.9E-14	RPS24/RPS15/RPS14/RPS11/RPS6/RPL35A/RPL32/RPL27A/RPL30/RPL24/RPL13/RPL10/RPL4/RPL3
establishment of protein localization to endoplasmic reticulum	14	BP	14/122	6.2E-14	RPS24/RPS15/RPS14/RPS11/RPS6/RPL35A/RPL32/RPL27A/RPL30/RPL24/RPL13/RPL10/RPL4/RPL3
establishment of protein localization to membrane	19	BP	19/122	7.4E-13	VPS37B/VAMP3/SDCBP/RPS24/RPS15/RPS14/RPS11/RPS6/RPL35A/RPL32/RPL27A/RPL30/RPL24/RPL13/RPL10/RPL4/RPL3/NACA/CDK5
protein targeting	21	BP	21/122	9.2E-13	VPS37B/SDCBP/RPS24/RPS15/RPS14/RPS11/RPS6/RPL35A/RPL32/RPL27A/RPL30/RPL24/RPL13/RPL10/RPL4/RPL3/NEDD4/NACA/M6PR/CDK5/CAT
protein localization to endoplasmic reticulum	14	BP	14/122	1.1E-12	RPS24/RPS15/RPS14/RPS11/RPS6/RPL35A/RPL32/RPL27A/RPL30/RPL24/RPL13/RPL10/RPL4/RPL3
viral transcription	14	BP	14/122	9.3E-12	RPS24/RPS15/RPS14/RPS11/RPS6/RPL35A/RPL32/RPL27A/RPL30/RPL24/RPL13/RPL10/RPL4/RPL3
viral gene expression	14	BP	14/122	3.2E-11	RPS24/RPS15/RPS14/RPS11/RPS6/RPL35A/RPL32/RPL27A/RPL30/RPL24/RPL13/RPL10/RPL4/RPL3
mRNA catabolic process	18	BP	18/122	3.7E-11	HNRNPR/SET/RPS24/RPS15/RPS14/RPS11/RPS6/RPL35A/RPL32/RPL27A/RPL30/RPL24/RPL13/RPL10/RPL4/RPL3/HNRNPM/HSPB1

nuclear-transcribed mRNA catabolic process	14	BP	14/122	8.6E-11	RPS24/RPS15/RPS14/RPS11/RPS6/RPL35A/RPL32/RPL27A/RPL30/RPL24/RPL13/RPL10/RPL4/RPL3
RNA catabolic process	18	BP	18/122	1.9E-10	HNRNPR/SET/RPS24/RPS15/RPS14/RPS11/RPS6/RPL35A/RPL32/RPL27A/RPL30/RPL24/RPL13/RPL10/RPL4/RPL3/HNRNPM/HSPB1
ribonucleoprotein complex biogenesis	18	BP	18/122	2E-09	LYAR/GAR1/RPL26L1/PRPF19/EIF3I/SNRPG/SNRPE/SNRPD1/RPS24/RPS15/RPS14/RPS6/RPL35A/RPL24/RPL10/RPL3/FBL/DDX3X
RNA splicing, via transesterification reactions with bulged adenosine as nucleophile	15	BP	15/122	3.4E-08	PHF5A/SF3B6/PRPF19/RALY/SF3B2/PPIE/HNRNPR/U2AF1/SNRPN/SNRPG/SNRPE/SNRPD1/SRSF2/PCBP1/HNRNPM
mRNA splicing, via spliceosome	15	BP	15/122	3.4E-08	PHF5A/SF3B6/PRPF19/RALY/SF3B2/PPIE/HNRNPR/U2AF1/SNRPN/SNRPG/SNRPE/SNRPD1/SRSF2/PCBP1/HNRNPM
RNA splicing, via transesterification reactions	15	BP	15/122	3.8E-08	PHF5A/SF3B6/PRPF19/RALY/SF3B2/PPIE/HNRNPR/U2AF1/SNRPN/SNRPG/SNRPE/SNRPD1/SRSF2/PCBP1/HNRNPM
ribosome biogenesis	13	BP	13/122	1.1E-07	LYAR/GAR1/RPL26L1/RPS24/RPS15/RPS14/RPS6/RPL35A/RPL24/RPL10/RPL3/FBL/DDX3X
RNA splicing	15	BP	15/122	5.9E-07	PHF5A/SF3B6/PRPF19/RALY/SF3B2/PPIE/HNRNPR/U2AF1/SNRPN/SNRPG/SNRPE/SNRPD1/SRSF2/PCBP1/HNRNPM
ribonucleoprotein complex assembly	10	BP	10/122	2.8E-06	PRPF19/EIF3I/SNRPG/SNRPE/SNRPD1/RPS15/RPS14/RPL24/RPL10/RPL3
ribonucleoprotein complex subunit organization	10	BP	10/122	3.7E-06	PRPF19/EIF3I/SNRPG/SNRPE/SNRPD1/RPS15/RPS14/RPL24/RPL10/RPL3
ribosome assembly	6	BP	6/122	4.7E-06	RPS15/RPS14/RPL24/RPL10/RPL3/DDX3X
regulation of viral life cycle	8	BP	8/122	8.3E-06	VPS37B/PPIE/TRIM28/BANF1/PPIB/IFI16/DDX3X/CD4
regulation of viral process	9	BP	9/122	1.2E-05	VPS37B/PPIE/TRIM28/BANF1/PPIB/IGF2R/IFI16/DDX3X/CD4
viral genome replication	7	BP	7/122	1.9E-05	PPIE/TRIM28/BANF1/PPIB/PCBP1/IFI16/DDX3X
regulation of symbiotic process	9	BP	9/122	1.9E-05	VPS37B/PPIE/TRIM28/BANF1/PPIB/IGF2R/IFI16/DDX3X/CD4
homotypic cell-cell adhesion	6	BP	6/122	2E-05	UBASH3B/VCL/CD99/ILK/HSPB1/COMP
nuclear transport	11	BP	11/122	2.1E-05	TRIM28/BANF1/U2AF1/SNRPG/SNRPE/SNRPD1/SRSF2/RPS15/NEDD4/HMGA1/CDK5
structural constituent of ribosome	15	MF	15/122	5.5E-12	RPL26L1/RPS24/RPS15/RPS14/RPS11/RPS6/RPL35A/RPL32/RPL27A/RPL30/RPL24/RPL13/RPL10/RPL4/RPL3
proteoglycan binding	4	MF	4/122	9.3E-05	THBS1/SDCBP/LRP1/COMP

collagen binding	5	MF	5/122	0.0001	THBS1/SPARC/PPIB/COMP/COL6A1
calcium-dependent protein binding	5	MF	5/122	0.00026	VPS37B/STMN2/S100A1/DMBT1/ANXA1
sulfur compound binding	8	MF	8/122	0.00037	THBS4/THBS1/SLIT1/LRP1/GSTP1/HBEGF/COMP/APOB
integrin binding	6	MF	6/122	0.0004	THBS4/THBS1/MFGE8/ILK/IGF2/COMP
extracellular matrix binding	4	MF	4/122	0.00056	SPOCK2/THBS1/SPARC/DMBT1
drug binding	5	MF	5/122	0.00065	PPIE/PYGL/PPIB/HMGB2/GSTP1
mRNA 5'-UTR binding	3	MF	3/122	0.00067	RPS14/MYH10/DDX3X
protein N-terminus binding	5	MF	5/122	0.00074	SLC9A3R1/BANF1/SDCBP/CSNK2A1/PARP1
glycosaminoglycan binding	7	MF	7/122	0.00092	SPOCK2/THBS4/THBS1/SLIT1/HBEGF/COMP/APOB
extracellular matrix structural constituent	6	MF	6/122	0.00094	THBS1/SPARC/MFGE8/COMP/COL6A1/CD4
heparin binding	6	MF	6/122	0.00094	THBS4/THBS1/SLIT1/HBEGF/COMP/APOB
phosphatidylethanolamine binding	2	MF	2/122	0.00232	PLTP/MFGE8
modified amino acid binding	4	MF	4/122	0.00306	CPNE1/THBS1/MFGE8/GSTP1
annealing activity	2	MF	2/122	0.00326	DDX3X/ANXA1
scavenger receptor activity	3	MF	3/122	0.00376	LRP1/DMBT1/CD5
amide binding	8	MF	8/122	0.00396	PPIE/ITM2A/PPIB/PLTP/LRP1/GSTP1/CD1C/CAT
peptide binding	7	MF	7/122	0.00454	PPIE/ITM2A/PPIB/LRP1/GSTP1/CD1C/CAT
heparan sulfate proteoglycan binding	2	MF	2/122	0.00495	LRP1/COMP
peroxidase activity	3	MF	3/122	0.005	HBA1/GSTP1/CAT
low-density lipoprotein particle binding	2	MF	2/122	0.00558	THBS1/PLTP
oxidoreductase activity, acting on peroxide as acceptor	3	MF	3/122	0.00615	HBA1/GSTP1/CAT
RNA stem-loop binding	2	MF	2/122	0.00626	MYH10/DDX3X
phosphatidylserine binding	3	MF	3/122	0.00678	CPNE1/THBS1/MFGE8
single-stranded DNA binding	4	MF	4/122	0.00707	SSBP1/PCBP1/HMGB2/ANXA1
cyclosporin A binding	2	MF	2/122	0.0077	PPIE/PPIB
adrenergic receptor binding	2	MF	2/122	0.0077	SLC9A3R1/NEDD4
cholesterol transfer activity	2	MF	2/122	0.0077	PLTP/APOB
sterol transfer activity	2	MF	2/122	0.00847	PLTP/APOB
cytosolic ribosome	17	CC	17/122	2.5E-19	RPL26L1/RPS24/RPS15/RPS14/RPS11/RPS6/RPL35A/RPL32/RPL27A/RPL30/RPL24/RPL13/RPL10/RPL4/RPL3/HBA1/DDX3X
ribosomal subunit	17	CC	17/122	2.4E-15	RPL26L1/RPS24/RPS15/RPS14/RPS11/RPS6/RPL35A/RPL32/RPL27A/RPL30/RPL24/RPL13/RPL10/RPL4/RPL3/HBA1/DDX3X
catalytic step 2 spliceosome	12	CC	12/122	2.4E-13	SF3B6/PRPF19/RALY/SF3B2/PPIE/HNRNPR/U2AF1/SNRPN/SNRPG/SNRPE/SNRPD1/HNRNPM

ribosome	17	CC	17/122	1E-12	RPL26L1/RPS24/RPS15/RPS14/RPS11/RPS6/RPL35A/RPL32/RPL27A/RPL30/RPL24/RPL13/RPL10/RPL4/RPL3/HBA1/DDX3X
focal adhesion	20	CC	20/122	1.3E-12	EHD3/PARVB/VCL/SDCBP/RPS15/RPS14/RPS11/RPL30/RPL4/RPL3/PPIB/CD99/LRP1/LIMS1/ILK/IGF2R/HSPB1/HMGA1/CAT/ANXA1
cell-substrate junction	20	CC	20/122	1.8E-12	EHD3/PARVB/VCL/SDCBP/RPS15/RPS14/RPS11/RPL30/RPL4/RPL3/PPIB/CD99/LRP1/LIMS1/ILK/IGF2R/HSPB1/HMGA1/CAT/ANXA1
cytosolic large ribosomal subunit	10	CC	10/122	2.5E-12	RPL26L1/RPL35A/RPL32/RPL27A/RPL30/RPL24/RPL13/RPL10/RPL4/RPL3
spliceosomal complex	14	CC	14/122	1.6E-11	PHF5A/SF3B6/PRPF19/RALY/SF3B2/PPIE/HNRNPR/U2AF1/SNRPN/SNRPG/SNRPE/SNRPD1/SRSF2/HNRNPM
U2 snRNP	7	CC	7/122	9.8E-11	PHF5A/SF3B6/SF3B2/SNRPN/SNRPG/SNRPE/SNRPD1
large ribosomal subunit	10	CC	10/122	2.9E-09	RPL26L1/RPL35A/RPL32/RPL27A/RPL30/RPL24/RPL13/RPL10/RPL4/RPL3
U2-type spliceosomal complex	9	CC	9/122	6.6E-09	PHF5A/SF3B6/PRPF19/SF3B2/PPIE/SNRPN/SNRPG/SNRPE/SNRPD1
cytosolic small ribosomal subunit	7	CC	7/122	1.6E-08	RPS24/RPS15/RPS14/RPS11/RPS6/HBA1/DDX3X
U12-type spliceosomal complex	6	CC	6/122	2.2E-08	PHF5A/SF3B6/SF3B2/SNRPG/SNRPE/SNRPD1
small ribosomal subunit	7	CC	7/122	3.9E-07	RPS24/RPS15/RPS14/RPS11/RPS6/HBA1/DDX3X
methylosome	4	CC	4/122	6.9E-07	SNRPG/SNRPE/SNRPD1/ERH
precatalytic spliceosome	6	CC	6/122	9.4E-07	PHF5A/SF3B6/SF3B2/SNRPG/SNRPE/SNRPD1
U4 snRNP	4	CC	4/122	9.9E-07	SNRPN/SNRPG/SNRPE/SNRPD1
vesicle lumen	12	CC	12/122	1E-06	PPIE/VCL/THBS1/SPARC/SDCBP/PYGL/IGF2/GSTP1/DDX3X/CAT/APOB/ADA
U2-type catalytic step 2 spliceosome	5	CC	5/122	1.1E-06	PRPF19/PPIE/SNRPG/SNRPE/SNRPD1
spliceosomal snRNP complex	7	CC	7/122	2.3E-06	PHF5A/SF3B6/SF3B2/SNRPN/SNRPG/SNRPE/SNRPD1
small nuclear ribonucleoprotein complex	7	CC	7/122	3.5E-06	PHF5A/SF3B6/SF3B2/SNRPN/SNRPG/SNRPE/SNRPD1
cytoplasmic vesicle lumen	11	CC	11/122	6.3E-06	PPIE/VCL/THBS1/SPARC/SDCBP/PYGL/IGF2/GSTP1/DDX3X/CAT/ADA
Sm-like protein family complex	7	CC	7/122	7.3E-06	PHF5A/SF3B6/SF3B2/SNRPN/SNRPG/SNRPE/SNRPD1
U5 snRNP	4	CC	4/122	9.7E-06	SNRPN/SNRPG/SNRPE/SNRPD1
ficolin-1-rich granule	7	CC	7/122	1.3E-05	PPIE/VCL/PYGL/PGM1/GSTP1/DDX3X/CAT
ficolin-1-rich granule lumen	7	CC	7/122	1.3E-05	PPIE/VCL/PYGL/PGM1/GSTP1/DDX3X/CAT

U2-type precatalytic spliceosome	5	CC	5/122	1.5E-05	PHF5A/SF3B2/SNRPG/SNRPE/SNRPD1
secretory granule lumen	10	CC	10/122	3.4E-05	PPIE/VCL/THBS1/SPARC/SDCBP/PYGL/IGF2/GSTP1/DDX3X/CAT
U1 snRNP	4	CC	4/122	8.1E-05	SNRPN/SNRPG/SNRPE/SNRPD1
clathrin-coated vesicle membrane	6	CC	6/122	9.7E-05	VAMP3/M6PR/IGF2R/HBEGF/CD4/APOB
Ribosome	15	KEGG	15/83	4.4E-11	RPL26L1/RPS24/RPS15/RPS14/RPS11/RPS6/RPL35A/RPL32/RPL27A/RPL30/RPL24/RPL13/RPL10/RPL4/RPL3
Coronavirus disease - COVID-19	16	KEGG	16/83	1.1E-09	RPL26L1/RPS24/RPS15/RPS14/RPS11/RPS6/RPL35A/RPL32/RPL27A/RPL30/RPL24/RPL13/RPL10/RPL4/RPL3/HBEGF
Spliceosome	12	KEGG	12/83	2.6E-08	PHF5A/SF3B6/PRPF19/SF3B2/PPIE/U2AF1/SNRPG/SNRPE/SNRPD1/SRSF2/PCBP1/HNRNPM
Malaria	5	KEGG	5/83	0.00015	THBS4/THBS1/LRP1/HBA1/COMP
ECM-receptor interaction	5	KEGG	5/83	0.00201	THBS4/THBS1/COMP/COL6A1/CD47
Focal adhesion	7	KEGG	7/83	0.00439	PARVB/VCL/THBS4/THBS1/ILK/COMP/COL6A1
Phagosome	6	KEGG	6/83	0.00454	TUBAL3/VAMP3/THBS4/THBS1/M6PR/COMP
Cholesterol metabolism	3	KEGG	3/83	0.01436	PLTP/LRP1/APOB
Amoebiasis	4	KEGG	4/83	0.02036	VCL/HSPB1/GNAQ/CD1C
Parathyroid hormone synthesis, secretion and action	4	KEGG	4/83	0.02309	SLC9A3R1/NACA/GNAQ/HBEGF
Tight junction	5	KEGG	5/83	0.02928	TUBAL3/SLC9A3R1/NEDD4/MYH10/CD1C
Carbon metabolism	4	KEGG	4/83	0.03	RGN/SUCLA2/CAT/ACO1
Citrate cycle (TCA cycle)	2	KEGG	2/83	0.03745	SUCLA2/ACO1
Pentose phosphate pathway	2	KEGG	2/83	0.03745	RGN/PGM1
Glyoxylate and dicarboxylate metabolism	2	KEGG	2/83	0.03745	CAT/ACO1
